# Quantum interference of single photons without optical superposition: Toward high resolution imaging in spatial and spectral domains

**DOI:** 10.1126/sciadv.aea9701

**Published:** 2026-04-22

**Authors:** Yunxiao Zhang, Liang Cui, Xueshi Guo, Guohui Kang, Wen Zhao, Qiqi Deng, Xuan Tang, Xiaoying Li, Chuanfeng Li, Z. Y. Ou

**Affiliations:** ^1^ The State Key Laboratory of Precision Measurement Technology and Instruments, College of Precision Instrument and Opto-Electronics Engineering, Tianjin University, Tianjin 300072, P. R. China.; ^2^ Department of Physics, City University of Hong Kong, 83 Tat Chee Avenue, Kowloon, Hong Kong, P. R. China.; ^3^ CAS Key Lab of Quantum Information, University of Science and Technology of China, Hefei 230026 P. R. China.

## Abstract

Observation of the universe demands telescopes with high resolution. In the optical band, traditional interference requires bringing interfering fields together, which limits the resolution due to the restricted length of baseline. Here we demonstrate the very long–baseline interferometer (VLBI) in optical band, where two interfering fields never met each other. In particular, we report the first quantum interference observation when the input of VLBI is single-photon state. Interference is recovered after measuring the amplitudes of photon fields and digitally processing the signals of quantum receivers. Moreover, we analyze interference in time and spectral domains for broadband thermal light input and show that the ultrahigh spectral resolution can improve the precision of radial velocity to 0.08 centimeters per second, which is 2 orders of magnitude better than that achievable at the current stage. Further, we apply the spectrally resolved interference in distinguishing two independent sources with angular resolutions beyond diffraction limit. Our investigations have a profound effect on the VLBI, quantum optics, and precision measurement.

## Teaser

We demonstrate a very long–baseline optical interferometry for ultrahigh-resolution imaging in both spatial and spectral domains.

## INTRODUCTION

The simultaneous quest for ever-higher spatial resolution and better wavelength coverage is the constant pursuit of astronomical imaging. Historically, astronomy began as an optical science. The discoveries Galileo made at the beginning of the 17th century by pointing his telescope at the sky revolutionized our understanding of the universe. Since the first radio astronomy observations in the 1930s (*1*), however, the field of high-resolution radio imaging has grown enormously (*2*–*4*). Currently, the highest angular resolution is realized by radio frequency (RF) interferometry. The very long–baseline interferometry (VLBI) technique being able to link radio telescopes at the locations separated by huge distance is the sharpest and the most sensitive tool for receiving radio signals from space (*4*). With VLBI virtual observatories that span the globe, the first high-resolution image of a black hole was recently synthesized. It is well known that the short wavelength of optical field naturally leads to improved resolution. However, a number of difficulties have prevented straightforward duplication of the VLBI in the optical domain (*3*, *5*–*10*).

On the other hand, astronomical images carry a lot of scientific information hidden within the frequency of electromagnetic radiation (*11*, *12*). A great deal of knowledge comes from analyzing the light as broken down into its spectrum. Beside investigating the temperature, density, and chemical composition, spectroscopy is successfully and largely used for studying the radial velocities of celestial objects via the Doppler shift, from which the motion of the universe can be estimated (*13*–*16*). So far, the resolution element (or minimum frequency resolution unit) achievable by current optical instruments for astronomy is several orders of magnitude larger than that of RF interferometers, which constitutes hindrances in better characterizing the absorption and emission lines in optical band.

Moreover, three-dimensional (3D) spectroscopy combining the information of imaging and spectrum together has attracted much attention in the past 40 years and has become a powerful tool to tackle astrophysical problems (*17*–*21*). The capability of mapping the distribution of celestial sources at different frequency will enable in-depth investigations of the material and processes involved in its formation and local environment (*21*). However, different from the high spatial resolution spectral imaging obtained by RF interferometry in a single exposure, the angular resolution of 3D spectroscopy in optical band has not surpassed the diffraction limit of single aperture yet.

The main difference between radio waves and optical waves lies in the detection method. The detection of RF wave depends on receiving antennas that preserve both phase and amplitude. Therefore, RF interferometer is able to coherently combine the signals of separate locations even if they are thousands of kilometers apart. In contrast, the most common detection method of optical wave is intensity measurement where the modulus square of the amplitude is measured and phase information is lost after detection. Therefore, the observation of optical interference requires bringing light together to within coherence length for optical superposition, which means that amplitude addition must be done before intensity detection. For example, the optical interferometric technique invented by Michelson (*22*) is supposed to have led to long-baseline astronomy and promises to increase resolution by orders of magnitude. However, the baseline size is currently limited to less than a few hundred meters because of the difficulty in balancing and stabilizing optical paths (*5*, *23*).

For quite a long time, it was thought that we cannot make direct measurement of the electric field in optical band because of the high oscillation frequency. The development in quantum optics around 1980s brought us the new detection technique of homodyne detection (HD), which is the optical equivalence of RF wave coherent detection and is known to measure the quadrature-phase amplitude of an optical field by mixing it with a strong local oscillator (LO) field serving as reference (*24*–*26*). Hence, different from intensity measurement, the measurement output of HD contains the phase information. If the complex amplitudes of optical fields can be directly measured and added thereafter, interference can occur although the interfering fields had never met at a common location. Moreover, different from the power detectors that accept optical radiation from a large number of spatial and temporal modes unless a specially designed optical filter can be used to reject unwanted modes, the HD is highly mode selective. The detected mode selected by the LO has exactly the same spatiotemporal and polarization profile as the LO. The unmatched modes will not contribute to the output of HD.

The discussion above is trivial in terms of wave aspect of optical fields, as is often done with RF interferometer. However, the energy of single quanta in optical bands is much higher than that in RF bands. For the electromagnetic radiation with a given intensity, the number of photons in optical band is much less. Hence, the quantum effect will play an important role when the photon number of starlight collected by telescopes is low. In particular, it becomes intriguing for single photons, which reflect the particle aspect of light (*27*). It was proposed by Burke (*28*) in the late 1960s that the RF VLBI may introduce a new facet in understanding the quantum interference. However, it is impractical to experimentally test the quantum interference of VLBI because of the overwhelming challenges in generating and detecting the single photons at RF (*3*, *29*).

Here, we report an interference experiment involving light fields in single-photon state, in which two interfering fields are well distinguished and have never met each other at detectors. We observe interference in the sum of the photocurrents after using quantum receivers (Q-RXs) to perform amplitude measurement at two spatially separated locations and digitally processing the photocurrents. The observation can be fully explained by the quantum theory of light, although there seems to be a dilemma about whether it is photon or electron that interferes here with itself in regard with the famous statement by Dirac on photon self-interference (*30*–*33*) because we do not add light amplitudes but the electronic currents, which are classical quantities and are not in quantum superposition. Moreover, the quantum interferometric method is a linear process and the input can be any optical field, which is a proof-of-principle demonstration of VLBI at optical frequency band. The optical signal processing functions including balancing paths and optical filtering can be performed at the electrical stage after detection. To illustrate that the VLBI removes the constraints and limitations of traditional optical interferometers, we then further investigate the interference fringe of VLBI in both time and spectral domains. Using broadband thermal light sources to mimic the emission of stars, we experimentally demonstrate a VLBI when the imbalance of two optical paths is over six orders magnitude larger than the coherence length. In addition, we show that spectrally resolved interference can be obtained over the spectrum and dynamic range of tens of terahertz and 54 dB, respectively, and the minimum frequency resolution unit is down to 1 kHz. The result indicates that the spectral resolution (R) is up to $10^{11}$, which implies that the radial velocity $v_{r}$ (related to the speed of light c through $v_{r}=c/R$) with precision down to level of 0.08 cm/s can be derived from such a high spectral resolution, and the demand for searching the earth like extrasolar planets and directly detecting the acceleration of the cosmic expansion can be satisfied (*15*, *16*, *34*). Furthermore, we manifest the prospects of the VLBI by demonstrating three applications, which cannot be accomplished by traditional interferometers. The first one is characterizing a polarization-dependent absorption line with ultrahigh spectral resolution when the input field is very weak; the second one is distinguishing two independent sources with spectrally resolvable angular resolutions breaking the diffraction limit of single aperture; and the third one is estimating the Doppler shift induced by the radial velocity down to 1±0.08 cm/s when a distant moving object is illuminated by thermal light. We believe that the quantum interferometric method, being able to simultaneously sample the information of wavelength, phase and amplitude, polarization, and direction of incident photons, opens the door for developing next generation interferometric instruments with ultrahigh resolution in both spatial and spectral domains, which will serve as powerful tools for diverse applications including but not limited to astronomy, space physics, precision measurement, and remote sensing.

## RESULTS

### Theory

**Fig. 1.**
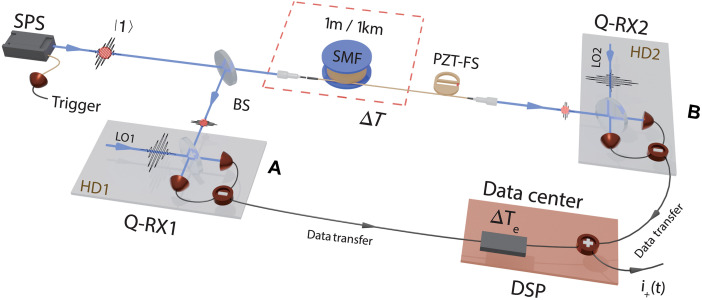
Conceptual representation for observing quantum interference of single-photon state without optical superposition. The single-photon state ∣1⟩ from SPS goes through a 50/50 BS, and two split fields independently propagate to spatially separated locations (A and B). At each location, the quadrature amplitudes of optical fields are respectively measured by the Q-RXs (Q-RX1 and Q-RX2), which are basically HDs (HD1 and HD2) with their own LOs (LO1 and LO2). The mode of strong LO1 and LO2 obtained from a laser well matches with that of input field. The photocurrent out of each Q-RX preserves the information of quadrature amplitude of its input. Wave superposition is realized by adding up the photocurrents of Q-RXs after transferring the data to a data center where the techniques of DSP is exploited. The relative optical delay Δ*T* for photon fields travel to locations A and B is introduced by SMF with variable length. When the relative phase between two optical fields is scanned by using a PZT-FS, interference can be recovered by properly introducing electronic delay $∆T_{e}$ in the photocurrent of Q-RX1 to ensure the satisfaction of the condition $∆T_{e}-$ Δ*T* = 0. In our experiments, the function of data center is realized by a DSO, in which the currents of two Q-RXs are gathered, stored, and postprocessed so that the functions of introducing adjustable electronic delay $∆T_{e}$ and recovering interference from the photocurrent addition $i_{+}\left(t\right)=\langle {\hat{i}}_{+}\left(t\right)\rangle$ are implemented.

We first look at a single-photon state ∣1⟩, which best describes the particle aspect of light field and usually does not carry any phase information (*27*). As shown in Fig. 1, we send the photon through a 50/50 beam splitter (BS) and produce an entangled state of single photon: $|\Psi \rangle =\frac{1}{\sqrt{2}}\left(|{0\rangle }_{1}|{1\rangle }_{2}+e^{i\theta }|{1\rangle }_{1}|{0\rangle }_{2}\right),$ where ∣0⟩ denotes the vacuum state, and θ is the relative phase difference between two optical paths. The two outputs of BS are then independently sent to separate locations (A and B). The path from BS to location B is very long, whereas the other path from BS to location A is short. We perform amplitude measurement to the optical fields at two locations by Q-RXs, Q-RX1 and Q-RX2, respectively. The interference pattern can be recovered when the photocurrents from two Q-RXs are transferred to a data center, in which the currents are properly processed and added up by exploiting digital signal processing (DSP) technique (*35*). It is straightforward to explain the phenomenon of recovered interference in terms of wave aspect of optical field. The Q-RX is basically an HD consisting of a 50/50 BS, two photodetectors, a strong LO, and a subtracter (see fig. S1 for details). The strong LO and weak input fields superpose on the BS, and the difference photocurrent is proportional to the quadrature amplitude of the input optical field (*24*–*26*, *36*–*37*). The current of HD is associated with the amplitude of input X(φ) through the relation $i_{\text{HD}}\propto |E|X\left(\varphi \right)$ with $X\left(\varphi \right)=a\left(t\right)e^{-i\varphi }+a^{*}\left(t\right)e^{i\varphi }$ and $E=|E|e^{-i\varphi }$ as the strong LO field. However, the contribution of vacuum state is not included in classical wave theory. On the other hand, quantum theory of light is a union of particle and wave and it should be able to fully explain the observed interference.

We place the single-photon state in a localized temporal mode with a pulse profile of f(t) (corresponding to pulse width or coherence time $\tau_{c}$), and the fields at locations A and B are represented by ${\hat{a}}_{1}f\left(t\right)$ and ${\hat{a}}_{2}\;f\left(t-\Delta T\right)$, respectively, where the optical delay Δ*T* ($∆T\gg \tau_{c}$) describes the time interval originated from the imbalance of two optical paths. To measure the amplitudes of the field at each location by Q-RXi (i=1,2), we need to have mode matched LO LOi: $E_{1}\;f\left(t\right)$ and $E_{2}\;f\left(t-∆T\right)$ with $E_{j}=|E_{j}|e^{i\varphi_{j}}$ (j=1,2). The current outputs of Q-RX1 and Q-RX2 are

$${\hat{i}}_{\text{HD}1}\left(t\right)\propto k\left(t\right)q_{1}|E_{1}|{\hat{X}}_{1}\left(\varphi_{1}\right)$$

$${\hat{i}}_{\text{HD}2}\left(t\right)\propto k^{\prime }\left(t-\Delta T\right)q_{2}|E_{2}|{\hat{X}}_{2}\left(\varphi_{2}\right)$$ (1)

where ${\hat{X}}_{j}$($\varphi_{j}$) ≡ ${\hat{a}}_{j}e^{-i\varphi_{j}}$ + ${\hat{a}}_{j}^{+}e^{-i\varphi_{j}}$ (*j* = 1, 2) is the quadrature-phase amplitude of each field, $q_{i}$ (i=1,2) is the electronic gain of ${\hat{i}}_{\text{HDi}}\left(t\right)$ determined by the circuits of HD and data processing, and k(t), $k^{\prime }\left(t\right)$ are the time response function of Q-RX1 and Q-RX2. For the sake of brevity, we assume that $q_{1}=q_{2}$. To ensure that the amplitude addition in the photocurrent will lead to wave superposition and interference, we introduce electronic delay $∆T_{e}$ in the output current of Q-RX1 to compensate Δ*T*. The addition of electronic currents can be written as

$$\begin{array}{c} {\hat{i}}_{+}\left(t\right)={\hat{i}}_{\text{HD}1}\left(t-∆T_{e}\right)+{\hat{i}}_{\text{HD}2}\left(t\right)\propto k\left(t-∆T_{e}\right)|E_{1}|{\hat{X}}_{1}\left(\varphi_{1}\right)\\ +k^{\prime }\left(t-\Delta T\right)|E_{2}|{\hat{X}}_{2}\left(\varphi_{2}\right) \end{array}$$ (2)

The visibility of interference showing up in the power of photo-current $\langle {\hat{i}}_{+}^{2}\left(t\right)\rangle$ with time integration due to long time average

$$\begin{array}{c} \int \mathrm{dt}{\langle {\hat{i}}_{+}^{2}\left(t\right)\rangle }_{|\Psi \rangle }\propto \frac{1}{2}\int \mathrm{dt}\left[k^{2}\left(t-∆T_{e}\right){|E_{1}|}^{2}{\langle {\hat{X}}_{1}^{2}\left(\varphi_{1}\right)\rangle }_{|\Psi \rangle }+k^{\prime 2}\left(t-\Delta T\right){\langle {\hat{X}}_{2}^{2}\left(\varphi_{2}\right)\rangle }_{|\Psi \rangle }\right]\\ +\int \mathrm{dtk}\left(t-∆T_{e}\right)k^{\prime }\left(t-∆T\right)|E_{1}||E_{2}|{\langle {\hat{X}}_{1}\left(\varphi_{1}\right){\hat{X}}_{2}\left(\varphi_{2}\right)\rangle }_{|\Psi \rangle }\\ \propto \left({|E_{1}|}^{2}+{|E_{2}|}^{2}\right)\left[1+V_{\mathrm{sp}}\text{cos}\left(\theta +\varphi_{1}-\varphi_{2}\right)\right] \end{array}$$ (3)

where

$$V_{\mathrm{sp}}\equiv \frac{|E_{1}||E_{2}|}{{|E_{1}|}^{2}+{|E_{2}|}^{2}}K\left(∆T-∆T_{e}\right)$$ (4)

refers to the visibility of interference with

$$K\left(∆T-∆T_{e}\right)=\frac{\int \mathrm{dtk}\left(t-∆T_{e}\right)k^{\prime }\left(t-∆T\right)}{\int \mathrm{dt}k^{2}\left(t\right)}$$ (5)

Equation 3 shows that the second-order correlation in amplitudes of the interfering fields $\langle {\hat{X}}_{1}\left(\varphi_{1}\right){\hat{X}}_{2}\left(\varphi_{2}\right)\rangle$ can be obtained even if the two fields, ${\hat{a}}_{1}f\left(t\right)$ and ${\hat{a}}_{2}\;f\left(t-\Delta T\right)$, have never met each other. The interference pattern will be observed when the relative phase (θ) between two paths is varied and the phase ($\text{Δφ}=\varphi_{1}-\varphi_{2}$) between LO1 and LO2 is fixed provided $K\left(∆T-∆T_{e}\right)\neq 0,$no matter how large the optical delay ∆T is. Assuming that two Q-RXs have identical time response [k(t) = $k^{\prime }\left(t\right)$], we always have $K\left(∆T-∆T_{e}\right)=1$ when $∆T-∆T_{e}=0$, and the highest visibility is one-half for $|E_{1}|=|E_{2}|$ due to the contribution of vacuum noise. Moreover, it should be emphasized that the interference is not between the field ${\hat{a}}_{1}f\left(t\right)$ $\left[{\hat{a}}_{2}\;f\left(t-\Delta T\right)\right]$ with the LO1 (LO2) because the phases of the single-photon state and LO are uncorrelated, which is fundamentally different from the detection in coherent optical communication (*38*). The interference stems from addition/superposition of the amplitudes of the two interfering fields, in which the role of LOs is to form HD for the amplitude measurement of the fields at locations A and B and is thus simply a bridge for the two fields to interfere. We notice that the observation of interference in Fig. 1 is analogous to the RF VLBI in the sense that the interference is recovered after detection (*3*). Therefore, the requirement on locking the phase difference of two LOs ($\varphi_{1}-\varphi_{2}$) is not as strict as that in an intensity correlation measurement (*39*) since this optical interferometer is a linear system and the imperfection induced by the phase drift of $\varphi_{1}-\varphi_{2}$ can be corrected in a certain extent by the combination of HD and DSP technique (*3*, *35*). Furthermore, in a real experiment, there exists extra vacuum noise introduced from losses, which results in a reduced visibility

$${V^{\prime }}_{\text{sp}}\equiv \frac{2\eta |E_{1}||E_{2}|}{\left(1+\eta \right)\left({|E_{1}|}^{2}+{|E_{2}|}^{2}\right)}K\left(∆T-∆T_{e}\right)$$ (6)

where η is the overall efficiency, including the efficiency of SPS, nonideal transmission, and detection efficiency.

### Experimental verification of the basic working principle

The detailed experimental schematic of Fig. 1 is introduced in Materials and Methods. The single-photon state with nearly single temporal mode is generated by heralding one of the twin photons from a pulse-pumped spontaneous four wave mixing in a piece of single-mode nonlinear fiber (*40*, *41*). The intensity correlation function of the heralded signal photons is measured to be $g^{\left(2\right)}=0.07\pm 0.005$, the heralding efficiency is about 50%, and the mode number is less than 1.3 (see Materials and Methods and the Supplementary Materials for details). The pump of single-photon source (SPS) is a laser pulse train with repetition rate of about 5.3 MHz. Thus, the interval between two adjacent single-photon pulses is $∆T_{s}=n\times ∆T_{p}$ (n≥1 is an integer) due to the feature of probabilistic generation (*42*), where $∆T_{p}=189$ ns is the interval between two adjacent pump pulses. The central wavelength, bandwidth, and coherence time ($\tau_{c}$) of the near transform-limited single-photon state are about 1553.3 nm, 1.1 nm, and 3 ps, respectively. The single-photon state input is split into two independently propagated fields by BS. One output of the BS is directly sent into Q-RX1, while the other, being coupled into a piece of single-mode fiber (SMF) to imitate the optical path imbalance and the separation distance of two locations, is measured by Q-RX2. The response time of each Q-RX is about $T_{R}=50$ ns [full width at half maximum (FWHM) of k(t)], which ensures that the single-photon events of two adjacent pulses are resolved. The strong LO (greater than $3.8\times 10^{8}$ photons per pulse) is synchronized with the pump of SPS, and the spectrum of pulsed LO is the same as that of single photons for optimum mode matching. The size of the photocurrent pulses out of Q-RX is proportional to the quadrature amplitude of the measured field. The photocurrents of two Q-RXs are then respectively converted into voltage and sent into a digital storage oscilloscope (DSO) triggered by the heralding signal of the single-photon events for DSP. In each set of measurement, the results of $4.85\times 10^{4}$ photocurrent pulses per second are recorded and processed. We evaluate the variance of amplitude measurement by extracting the peak of each current pulse (see fig. S3 for details).

In the process of observing interference, the phase θ between two optical paths is slowly scanned by changing the voltage applied on a piezoelectric transducer–driven fiber stretcher (PZT-FS), while the phase between LO1 and LO2 ($∆\varphi =\varphi_{1}-\varphi_{2}$) is managed to be constant to simplify the algorithm used in DSP. The photocurrent of each Q-RX measures the quadrature amplitude of single photons attenuated by the 50/50 BS. The quadrature amplitude measured by one Q-RX can be both positive and negative (see fig. S3 for details), whose average is the same as vacuum but variance is higher than that of vacuum (*43*). Interference can be observed from the current addition of two Q-RXs when the electronic delay $∆T_{e}$ is introduced in the output of Q-RX1 to ensure the satisfaction of $∆T-∆T_{e}=0$.

**Fig. 2.**
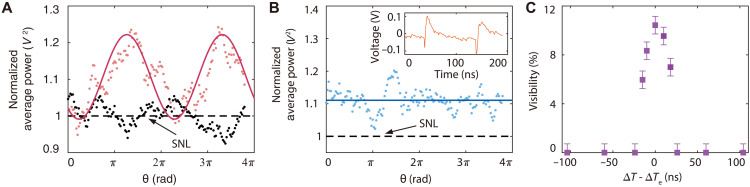
Experimental results when the split fields of single photons are sent to separate locations and the relative phase θ between two optical paths is slowly scanned. Normalized average power of current $\langle {\hat{i}}_{+}^{2}\left(t\right)\rangle$ (in terms of voltage) when the relation between electronic delay and optical delay is (**A**) $∆T_{e}=∆T=5$ ns and (**B**) $∆T_{e}-∆T=104$ ns, respectively. The pink curve and blue straight line, indicating the visibility ${V^{\prime }}_{\text{sp}}$ is (10.4±0.7)% and nonobservable, are the fitting and average of data points represented by the pink and blue dots, respectively. The black dots in (A) obtained by blocking the inputs of Q-RXs represent the variance of vacuum. The averages of vacuum fluctuation are expressed by the black dashed lines, indicating the SNL. The normalization is done to the SNL [${\langle {\hat{i}}_{+}^{2}\rangle }_{\mathrm{SNL}}$ = $1.39\times 10^{-5}$ *V*^2^ for (A) and ${\langle {\hat{i}}_{+}^{2}\rangle }_{\mathrm{SNL}}$ = $1.05\times 10^{-5}$ *V*^2^ for (B)]. The inset in (B) demonstrates the profile of photocurrent $\langle {\hat{i}}_{+}\left(t\right)\rangle$ when $∆T_{e}-∆T=104$ ns. Two electric current pulses separated by 104 ns (orange trace) illustrate the response functions $k^{\prime }\left(t-∆T\right),$ and $k\left(t-∆T_{e}\right)$ of two the Q-RXs are nonoverlapping, leading to ${V^{\prime }}_{\text{sp}}=K\left(∆T-∆T_{e}\right)$=0 (see Eqs. 5 and 6). (**C**) Visibility of the recovered interference as a function of $∆T-∆T_{e}$. The highest visibility is achieved when $∆T_{e}-∆T=0$. The visibility decreases with the increase of $|∆T_{e}-∆T|$ and approaches to 0 when $|∆T_{e}-∆T|>25$ ns. In the experiment, the coherence time of single-photon state is $\tau_{c}\approx 3$ ps, and the response time of each Q-RX ($T_{R}\approx 50$ ns) is fast enough to resolve the single-photon events in adjacent pulses $∆T_{s}=n\times 189$ ns (n≥1 is an integer).

We first verify the working principle. Without loss of the generality, we perform measurement when the optical delay ∆T=5 ns is introduced by a 1-m-long SMF. Figure 2A plots the data when the electronic delay well balances the optical delay, i.e., $∆T_{e}=∆T=5$ ns, where each data point represents the amplitude variance evaluated from 130 pulses of electric current. Clearly, the amplitude variance (pink dots) measured by the average power of current $\langle {\hat{i}}_{+}^{2}\left(t\right)\rangle$ varies with the phase scanning, showing an interference pattern. As a comparison, we also measure the amplitude variance of vacuum (black dots) by blocking the input of each Q-RX, which corresponds to the shot noise level (SNL), and normalize the measurement results to it. The pink solid curve is the fitting to a sine function for the input of single photons, showing that the visibility of interference fringe is (10.4±0.7)%, while the black dashed line is the average of SNL. Taking the factor of total detection efficiency ∼16% into account (see Materials and Methods for detail), the result is slightly lower than the theoretical expectation of about 13.7% because the circuits of two high efficiency Q-RXs have not been specifically designed to make their response function identical.

The blue dots in Fig. 2B are the measurement results when $∆T_{e}-∆T=104$ ns. In this case, all data points are above SNL and the average of the data (blue solid line) is above SNL (black dash line) by about 0.4 dB, showing that no interference is observable. When the photocurrent pulses from two Q-RXs are added up, they become distinguishable (see the inset in Fig. 2B) because $∆T_{e}$ is not properly set to compensate ∆T. The phenomena agree with the theory prediction of Eqs. 5 and 6, in which nonoverlap of the current pulses with temporal function determined by $k\left(t-∆T_{e}\right)$ and $k^{\prime }\left(t-∆T\right)$ leads to $K\left(∆T-∆T_{e}\right)=0={V^{\prime }}_{\text{sp}}$ .

To better understand the time dependence of interference, we process the data to extract the visibility ${V^{\prime }}_{\text{sp}}$ when $∆T_{e}$ is finely tuned in the vicinity of $∆T_{e}-∆T=0$. As shown in Fig. 2C, the visibility decreases with the increase of $|∆T_{e}-∆T|$ and approaches to 0 when $|∆T_{e}-∆T|>25$ ns, which is determined by the response time ($T_{R}\approx$ 50 ns) of Q-RX and is qualitatively consistent with the width of function $K\left(∆T-∆T_{e}\right)$ (Eq. 5). The result verifies that the FWHM of ${V^{\prime }}_{\text{sp}}$ determined by the time response function of Q-RXs is irrelevant to the coherence time $\tau_{c}$ of broadband input as long as $\tau_{c}<T_{R}$ (*37*). The necessary condition of measuring the second-order correlation of interfering fields $\langle {\hat{X}}_{1}\left(t,\varphi_{1}\right){\hat{X}}_{2}(t,\varphi_{2})\rangle =|\gamma |\text{cos}\left(\theta +\varphi_{1}-\varphi_{2}\right)$ from current addition $\langle {\hat{i}}_{+}\left(t\right)\rangle$ is $∆T-∆T_{e}=0$, where the visibility is the highest.

### Break through the constraints of traditional optical interferometers

The input of the scheme in Fig. 1 is not limited to the single-photon state but can be any optical fields, including the single mode, multimode, continuous wave (CW), and pulsed fields. Using the thermal field as input to mimic the emission of stars, we carry out two sets of experiments to clarify the quantum interferometric method of removal of constrains of the traditional optical interferometer.

#### VLBI with no restriction of optical path imbalance

We first demonstrate that the restriction of extending baseline caused by the difficulty of balancing paths vanishes since the requirement of balancing optical paths is eliminated. In the experiment, the input of the single-photon state is replaced with a thermal field, which is simply realized by performing measurement when the trigger signal of heralding field in Fig. 1 is removed. Traditionally, the measurement of second-order correlation $\gamma \left(\tau \right)=\frac{\langle {\hat{E}}_{1}^{†}(t){\hat{E}}_{2}\left(t+\tau \right)\rangle }{\sqrt{I_{1}I_{2}}}=|\gamma \left(\tau \right)|e^{i\theta }$ with $|\gamma \left(\tau \right)|=e^{-\frac{\tau^{2}}{\tau_{c}^{2}}}$ requires bringing two interfering fields ${\hat{E}}_{j}\left(t\right)={\hat{a}}_{j}f\left(t\right)$ (j=1,2) together and matching the length of optical paths to ensure that the delay is $\tau <\tau_{c}$. Here, we further enlarge the distance between the spatially separated locations A and B by increasing the length of SMF to 1 km, which means that the delay ∆T induced by the imbalance of two optical paths is more than six orders magnitude larger than the coherence time.

In this experiment, the mode number of thermal state input is nearly in single temporal mode (see Materials and Methods for details) (*44*). We know the variance of amplitude $\langle {\hat{X}}^{2}\rangle =2\bar{n}+1$ for thermal light of average photon $\bar{n},$ with 1 denoting the vacuum noise. It is straightforward to figure out the expression for the visibility of interference (*37*)

$$V_{t}=K\left(∆T-\Delta T_{e}\right)\frac{2\eta^{\prime }{\bar{n}}^{\prime }}{2\eta^{\prime }{\bar{n}}^{\prime }+1}$$ (7)

when $|E_{1}|=|E_{2}|,$where ${\bar{n}}^{\prime }$ is the average photon number per pulse for the input of each Q-RX, and $\eta^{\prime }$ is the detection efficiency determined by the mode matching and quantum efficiency of Q-RX. When $∆T-\Delta T_{e}=0$, the visibility takes the maximum and is related to the second-order correlation through the relation $V_{t}=|\gamma \left(0\right)|\frac{2\eta^{\prime }{\bar{n}}^{\prime }}{2\eta^{\prime }{\bar{n}}^{\prime }+1}$. Equation 7 shows that the influence of vacuum noise will become negligible when the average photon number $\eta^{\prime }{\bar{n}}^{\prime }$ is much greater than 1.

**Fig. 3.**
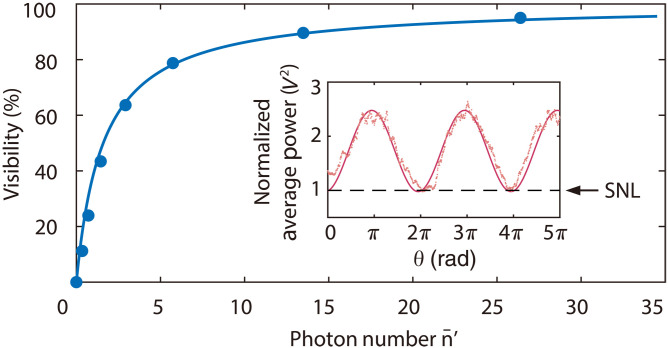
Interference observed by using thermal state input to mimic the photon statistics of light emitted by stars and by changing the length of SMF in Fig. 1 to 1 km to imitate the optical path imbalance. The main plot shows the observed maximum visibility versus the average photon number ${\bar{n}}^{\prime }$ of the thermal field. The data points (blue dots) well fit Eq. 7 with $K\left(∆T-\Delta T_{e}\right)=1$ and $\eta^{\prime }\approx 0.3$ (blue curve). The inset displays the normalized average power of current $\langle {\hat{i}}_{+}^{2}\left(t\right)\rangle$ (in terms of voltage) versus the phase difference θ between two optical paths when the average photon number of field measured by each Q-RX is $\eta^{\prime }{\bar{n}}^{\prime }\approx$ 0.4 photons per pulse and $∆T_{e}-∆T=0$. The pink curve showing the visibility of (43.4±0.6)% is the fitting of the data (pink dots). Black dashed line represents the SNL, and the normalization is done to the SNL (${\langle {\hat{i}}_{+}^{2}\rangle }_{\mathrm{SNL}}$ = $2.77\times 10^{-5}$ V^2^). In this experiment, the coherence time of thermal field input is $\tau_{c}\approx 3$ ps.

Figure 3 shows the optimum visibility (blue dots) observed by varying the average photon number ${\bar{n}}^{\prime }$. Obviously, the visibility increases with ${\bar{n}}^{\prime }$. The visibility is about 94% for ${\bar{n}}^{\prime }\approx 27$ (see Materials and Methods for details). The best fitting (blue curve) of the data is obtained when the efficiency in Eq. 7 is $\eta^{\prime }\approx 0.3$, which is consistent with the experimental parameters. To illustrate the typical feature of interference fringe, in the inset of Fig. 3, we demonstrate the interference pattern when $\eta^{\prime }{\bar{n}}^{\prime }\approx 0.4$ and $∆T_{e}-∆T=0.$ The pink curve is the fitting of data (pink dots) to a sine function, indicating that the visibility is up to (43.4±0.6)%. Moreover, we note that for the interference patterns observed with the weak input field, the level of peaks increases with ${\bar{n}}^{\prime }$, but level of the troughs is the same as that of SNL (black dashed line in inset). In other word, the visibility is vacuum-noise limited.

#### VLBI with ultrahigh spectral resolution

**Fig. 4.**
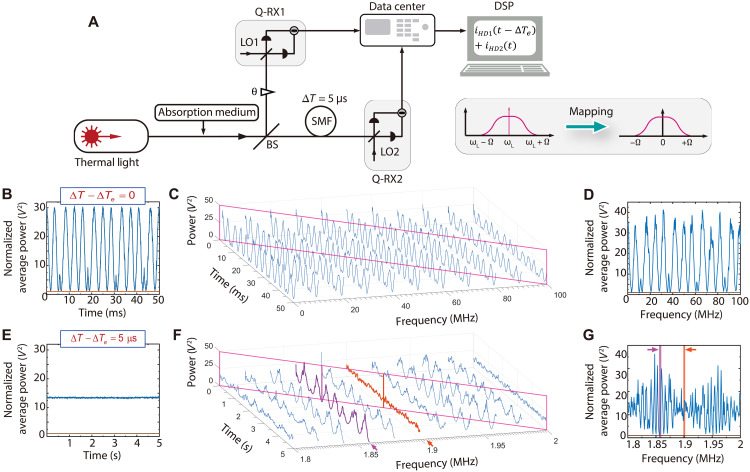
Observation of the interference patterns with ultrahigh spectral resolution. (**A**) Experimental scheme realized by replacing the input of Fig. 1 with a CW broadband thermal light to mimic star emission. The strong LO of each Q-RX comes from a single-frequency laser at $\omega_{l}$. The inset illustrates that Q-RX coherently maps the optical frequency $\omega_{l}\pm \Omega$ into RF range ±Ω with $|\Omega |<1/T_{R}$. The interference patterns are observed by varying the algorithm and electronic delay $\Delta T_{e}$ in DSP when the phase θ is scanned. When $|∆T-\Delta T_{e}|=0$, the interference in (**B**) time and (**C**) frequency domains, respectively, represented by the normalized average power and power spectrum of current addition, $\langle {\hat{i}}_{+}^{2}\left(t\right)\rangle$ and S(Ω). (**D**) Power spectrum S(Ω) extracted from the cross section (pink parallelogram) in (C) clearly exhibits that the visibility $V_{\Omega }$ always takes the optimum value. $V_{\Omega }$ of each frequency component ($\omega_{l}\pm \Omega$) in (C) is the same as the visibility $V_{t}\approx$93% in (B). When $|∆T-\Delta T_{e}|=5$ μs $\gg T_{R}$, (**E**) the interference is unobservable from $\langle {\hat{i}}_{+}^{2}\left(t\right)\rangle$; however, (**F**) the interference with frequency-dependent visibility can be observed from S(Ω) when the resolution bandwidth ∆f in DSP is narrow enough. (**G**) Power spectrum S(Ω) extracted from the cross section (pink parallelogram) in (F) manifests that $V_{\Omega }$ highly depend on frequency. In particular, the point at the frequency Ω=1.852 (1.901) MHz marked by the purple (orange) arrow is originated from the purple (orange) trace in (F), where the visibility is ~93% (unobservable). The results agree with the predictions of Eqs. 8 and 10. Brown lines in (B) to (G) represent the SNL. In the experiment, ∆T= 5 μs, $\lambda_{l}=c/\omega_{l}$ = 1550.1 nm, $T_{R}\approx 10$ ns, ∆f for (C) and (F) are 200 and 0.8 kHz, respectively, and average photon number of input is $\eta^{\prime }{\bar{n}}^{\prime }\approx 7$.

We then demonstrate that the quantum interferometric method works for the input of CW broadband thermal light as well, as shown in Fig. 4. The experimental scheme in Fig. 4A is similar to that in Fig. 1, but the input is CW thermal field with spectrum covering the wavelength range of 1200 to 1620 nm (see Materials and Methods for details). The strong LOS (LO1 and LO2) $E_{j}\left(t\right)=|E_{j}|e^{i\varphi_{j}-i\omega_{l}t}$ (j=1,2) of Q-RXs are split from a CW laser at frequency $\omega_{l}$. In the frequency domain, the Q-RX is essentially a baseband receiver, which coherently maps an optical field in the range of $\omega_{l}\pm \Omega$ into RF domain (see the inset of Fig. 4A), and the range of RF Ω is determined by the frequency response function of Q-RX $k\left(\Omega \right)=\int d\tau k\left(\tau \right)e^{i\text{Ωτ}}$. The Q-RX only measures the quadrature amplitude of the frequency component well matched with LO, and the strong LO amplifies the quadrature amplitude of selected weak input field (see Eq. 1) by ~40 dB. As a result, the electronic noise of Q-RX can be neglected, and the influence of other frequency components of the weak input is negligible. Therefore, the quantum interferometer is especially convenient when high spectral resolution is needed because an arbitrarily narrow frequency band in RF can be easily isolated and the narrow optical filter inevitably introducing loss is no longer required.

For the CW broadband thermal field input $\hat{E}\left(t\right)=\frac{1}{\sqrt{2\pi }}\int d\omega \hat{a}\left(\omega \right)e^{-i\omega t}$ satisfying the commutation relation $\left[\hat{a}\left(\omega \right),{\hat{a}}^{†}\left(\omega^{\prime }\right)\right]=\delta \left(\omega -\omega^{\prime }\right),$ the power of current addition in time domain is expressed as (*37*)

$$\langle {\hat{i}}_{+}^{2}\left(t\right)\rangle ={\langle {\hat{i}}_{+}^{2}\rangle }_{\mathrm{SNL}}\left(2\eta^{\prime }{\bar{n}}^{\prime }+1\right)\left[1+V_{t}\text{cos}\left(\theta +\varphi_{1}-\varphi_{2}\right)\right]$$ (8)

where ${\langle {\hat{i}}_{+}^{2}\rangle }_{\mathrm{SNL}}\propto \left({|E_{1}|}^{2}+{|E_{2}|}^{2}\right)\;\Delta B$ with $\Delta B=\frac{1}{2\pi }\int d\Omega {|k\left(\Omega \right)|}^{2}$ denoting the frequency bandwidth of Q-RX is the SNL (see Materials and Methods for details), ${\bar{n}}^{\prime }=\langle {\hat{a}}^{†}\left(\omega \right)\hat{a}\left(\omega \right)\rangle$ is the average photon number (in the unit of photons per Hz) of input, $\eta^{\prime }$ is the detection efficiency of Q-RX, and the expression of visibility $V_{t}$ is the same as Eq. 7. On the other hand, the optical field at frequency $\omega_{l}\pm \Omega$ can be measured and analyzed by applying digital filter centering at Ω in DSP. In this situation, the current addition in frequency domain is written as

$${\hat{i}}_{+}\left(\Omega \right)=k\left(\Omega \right)e^{-i\text{ΩΔ}T_{e}}|E_{1}|{\hat{X}}_{1}\left(\Omega \right)+k\left(\Omega \right)e^{-i\text{ΩΔ}T}|E_{2}|{\hat{X}}_{2}\left(\Omega \right)$$ (9)

where ${\hat{X}}_{j}\left(\Omega \right)\propto {\hat{a}}_{j}\left(\omega_{l}-\Omega \right)e^{-i\varphi_{j}}+{\hat{a}}_{j}^{†}\left(\omega_{l}+\Omega \right)e^{i\varphi_{j}}$ (j=1,2) is quadrature amplitude measured by the two Q-Rxs. Accordingly, it is straightforward to derive the power spectrum of the current addition

$$S\left(\Omega \right)=\langle {\hat{i}}_{+}^{†}\left(\Omega \right){\hat{i}}_{+}\left(\Omega \right)\rangle =S_{\mathrm{SN}}\left(\Omega \right)\left(2\eta^{\prime }{\bar{n}}^{\prime }+1\right)[1+V_{\Omega }\text{cos}\left(\theta +\varphi_{1}-\varphi_{2}\right)]$$ (10)

with

$$V_{\Omega }=|\text{cos}2\text{πΩ}\left(∆T-\Delta T_{e}\right)|\frac{2\eta^{\prime }{\bar{n}}^{\prime }}{2\eta^{\prime }{\bar{n}}^{\prime }+1}$$ (11)

referring to the visibility of the field $\hat{E}\left(t\right)={\hat{a}}_{j}\left(\omega_{l}+\Omega \right)e^{-i\left(\omega_{l}+\Omega \right)t}+{\hat{a}}_{j}\left(\omega_{l}-\Omega \right)e^{-i\left(\omega_{l}-\Omega \right)t}$, where $S_{\mathrm{SN}}\left(\Omega \right)\propto \left({|E_{1}|}^{2}+{|E_{2}|}^{2}\right){|k\left(\Omega \right)|}^{2}$ is the SNL, and ${\bar{n}}^{\prime }=\langle {\hat{a}}^{†}\left(\omega_{l}\pm \Omega \right)\hat{a}\left(\omega_{l}\pm \Omega \right)\rangle$ denotes the average photon number at frequency $\omega_{l}\pm \Omega$. Note that S(Ω) can be obtained from the current addition in time domains through the Fourier transformation, i.e., $S\left(\Omega \right)={\left|F\left[{\hat{i}}_{+}\left(t\right)\right]\right|}^{2}=\frac{1}{T}\int \mathrm{dt}\int d\tau \langle {\hat{i}}_{+}\left(t\right){\hat{i}}_{+}\left(t+\tau \right)\rangle e^{i\text{Ωτ}}$, where T is the time period for taking data. When $∆T-\Delta T_{e}\gg T_{R}$, the visibility of time domain interference becomes $V_{t}=0$ (Eqs. 5 and 7), but the frequency dependent visibility $V_{\Omega }$ always exists as long as the resolution bandwidth of digital filter applied in spectral analysis is narrow enough. On the other hand, we have $V_{\Omega }\equiv$ $V_{t}$ when $∆T-\Delta T_{e}=0$ and $\eta^{\prime }{\bar{n}}^{\prime }$ stays constant within the frequency response range of Q-RX. In principle, the full-spectrum information of the broadband input can be obtained by two methods. One is to simultaneously measure the quadrature amplitudes of the different frequency components by an array of Q-RXs after mixing the input with the multiple frequency LO come from a laser frequency comb and exploiting the wavelength division multiplexing technique to separate the frequency components selected by the individual lines of frequency comb (*45*). The other is tuning the wavelength of single-frequency LO to sequentially select and measure the different frequency components. The minimum frequency resolution unit is determined by the bandwidth of electrical filter applied in DSP. Similar to the RF interferometry (*3*), there is no fundamental limitation to the frequency resolution unit, which is a trade-off between the sampling rate and time resolution.

Figure 4 (B to G) demonstrates the recovered interference in time and frequency domains when the optical delay is fixed at ∆T=5 μs and the relative phase θ is properly scanned. In this experiment, the wavelength, linewidth, and power of LO are 1550.1 nm, 1 kHz, and ~0.8 mW, respectively. The response time of each Q-RX is $T_{R}\approx 10$ ns, and the average photon number of weak input is fixed at $\eta^{\prime }{\bar{n}}^{\prime }\approx 7$. When $|∆T-\Delta T_{e}|=0$, the interference with visibility of about $V_{t}\approx$ 93% is recovered from the average power of current addition $\langle {\hat{i}}_{+}^{2}\left(t\right)\rangle$ (Fig. 4B). During the process of data acquisition, the sampling rate and sampling time of DSP is 1.25 GHz and 50 ms (see Materials and Methods for details). Using the algorithm of fast Fourier transform (FFT) in DSP, we arrive at the power spectrum S(Ω) (Fig. 4C), in which the interference pattern for each frequency component $\omega_{l}\pm \Omega$ with Ω=n×∆f MHz (0≤n≤500 and ∆f=200 kHz) is obtainable. The power spectrum S(Ω) in Fig. 4D achieved by scanning the frequency and phase θ simultaneously is equivalent to the cross section labeled by pink parallelogram in Fig. 4C, which explicitly exhibits the visibility $V_{\Omega }$ in the whole frequency response range 0<Ω<100 MHz is the same as that in Fig. 4B. When $|∆T-\Delta T_{e}|=5$ μs, interference fringe is unobservable from $\langle {\hat{i}}_{+}^{2}\left(t\right)\rangle$ (Fig. 4E) because the nonoverlap of the currents $k\left(t-∆T_{e}\right)$ and k(t−∆T) leads to $V_{t}=K\left(∆T-\Delta T_{e}\right)=0$. In this case, interference fringe in the frequency range of $|\Omega |<1/T_{R}$ cannot be observed from S(Ω) as well if the resolution bandwidth (RBW) ∆f is still 200 kHz (see Materials and Methods for details). However, the interference with frequency dependent visibility can be observed from S(Ω) (Fig. 4F) when the RBW is reduced by increasing the sampling time and decreasing the frequency analyzing range in DSP. Figure 4F displays a representative part of S(Ω), from which the interference pattern for each frequency component $\omega_{l}\pm \Omega$ with Ω=1.8+n×∆f MHz (0≤n≤250 and ∆f=0.8 kHz) can be achieved. The power spectrum S(Ω) in Fig. 4G is extracted from the cross section labeled by pink parallelogram in Fig. 4F, which clearly illustrates that $V_{\Omega }$ varies with Ω. The data point at Ω=1.852 (1.901) MHz, marked out by the purple (orange) line and the purple (orange) arrow in Fig. 4G, is originated from the purple (orange) trace in Fig. 4F, where the visibility of interference fringe is ~93% (unobservable) due to the frequency dependent constructive (destructive) interference. We note that for the data in Fig. 4, the average powers are normalized to SNL (see Materials and Methods for details), and the visibilities of interference in time and frequency domain agree with predictions of Eqs. 8 and 10, respectively.

The results in Fig. 4 indicate that the minimum frequency resolution unit ∆f of the interferometer can be smaller than 1 kHz, and part of input spectral information deduced from the spectrally resolved interference is distorted or even missing unless $|∆T-\Delta T_{e}|=0$. Notice that the spectral resolution ($R=\frac{\omega_{l}}{\Delta f}$) is up to $10^{11}$, which is more than five orders magnitude higher than that achievable by current optical instruments for astronomy (*13*) and still has space to be further increased. Moreover, the signal-to-noise ratio of the information read from the interference fringe can be improved by properly multiplexing the spectrally resolved interference patterns (see fig. S7 and the Supplementary Materials for details). Furthermore, the signal size measured by Q-RX linearly increase with $\eta^{\prime }{\bar{n}}^{\prime }$ when $\eta^{\prime }{\bar{n}}^{\prime }$ is in the range of 0.05 to $1.6\times 10^{4}$ (see fig. S6 and the Supplementary Materials for details), illustrating that the linear dynamic range is about 54 dB. Hence, the quantum interferometric method being able to measure the second order coherence function with ultrahigh spectral resolution will be a powerful tool for spectral analysis and 3D spectroscopy.

### Implement the missions impossible for traditional optical interferometers

#### Characterization of the polarization-dependent absorption line

**Fig. 5.**
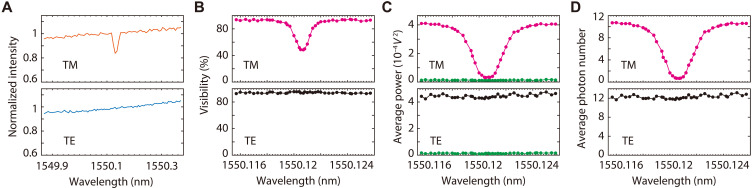
Characterization of the polarization-dependent absorption line. (**A**) The spectra of two orthogonal polarization modes (TM and TE) directly measured by an OSA indicate that the absorption-induced intensity reduction does not exist in TE mode (blue trace), the absorption line in TM mode is centering at about 1550.12 nm, but the values of absorption width and depth cannot be reliably read out (orange trace) since the spectral resolution of OSA is only 0.02 nm. (**B** to **D**) The detailed information of the absorption line measured by using the spectrally resolved interference of the quantum interferometer. (D) The average photon number $\eta^{\prime }{\bar{n}}^{\prime }$ versus the wavelength of input field in TM (pink dots) and TE (black dots) modes are deduced from (B) the visibilities and (C) size of interference fringes, obtained by varying the wavelength of LO in polarization modes of TE and TM, respectively. The absorption-induced reduction is obvious (unobservable) from the data represented by pink (black) dots. The spectrum obtained from the interferometric method indicates that the peak, depth, and width of the absorption line are 1550.1206 nm, above 90% and 0.0024 nm (300 MHz), respectively. The green circles in (C) representing the levels of the troughs of interference fringes overlap with the SNL. In the experiment, the wavelength and polarization of LO can be varied, $|∆T-\Delta T_{e}|=0$, and bandwidth of electrical filter used in DSP is 10 MHz.

We characterize the polarization-dependent absorption line with high spectral resolution by exploiting the spectrally resolvable interference pattern obtained from the quantum interferometer. In the experiment, a polarization-dependent absorption medium (see fig. S9 and the Supplementary Materials for details) is inserted into the scheme in Fig. 4A. Figure 5A exhibits a small part of spectra directly measured by placing a polarizer and an optical spectrum analyzer (OSA) right after the absorption medium. The spectra of two orthogonal polarization modes indicate that the absorption is polarization dependent since the absorption-induced intensity reduction is not observable in transverse electric (TE) mode (blue trace). The absorption peak at about 1550.12 nm is obvious in the spectrum of transverse magnetic (TM) mode (orange trace), but the information of absorption width (0.02 nm) and depth (~10%) read from Fig. 5A is not accurate and reliable because the spectral resolution of the OSA is only 0.02 nm. In contrast, the detailed information of the absorption line can be obtained by the interferometer, in which a frequency tunable laser serves as LOs. When the polarization of LO is parallel and orthogonal with TM mode of the absorption medium, respectively, we tune the wavelength of LO from 1549.8 to 1550.5 nm with a step of ~$3\times 10^{-4}$ nm (see fig. S10 for details). Moreover, the condition $|∆T-\Delta T_{e}|=0$ holds to ensure that the full spectral information of input is accessible. For each setting of LO, we scan the relative phase of two optical paths and acquire the visibility, fringe size, and SNL from the peaks and troughs of interference patterns. As shown in Fig. 5 (B and C), the visibility and fringe size vary with wavelength and polarization of LO. The wavelength dependent dip is unobservable (black dots) when the polarization is orthogonal with TM mode. When the polarization is parallel to TM mode, the visibility and fringe size (pink dots) take the minimum at the absorption peak of 1550.1206 nm. According to Fig. 5 (B and C), the average number of weak input field as a function of wavelength can be deduced by taking the influence of vacuum fluctuation into account (see the Supplementary Materials for details). The spectra of input at two orthogonal polarizations in Fig. 5D clearly illustrate the feature of the absorption spectrum. The accurate values of absorption width and depth, 0.0024 nm (~300 MHz) and >90%, can be read out from the data (pink dots) in Fig. 5D. Note that in Fig. 5 (B to D), the bandwidth of electrical filter in DSP is chosen to be 10 MHz. In this case, the algorithm in DSP is sample, and the complex coherence function of two interfering field at different wavelength is obtained by tuning the wavelength of LOs. However, a finer frequency resolution down to the kilohertz level is achievable by performing a more complete measurement and applying more sophisticated algorithm in DSP (*3*). Moreover, the information of absorption spectrum can be obtained from the power current of individual Q-RX as well if the background noise is negligible. However, the current addition of two Q-RXs is a result of the spectrally resolved complex visibility, which not only supplies a better signal-to-noise ratio for weak input but provides a powerful tool for resolving the structure of light sources, as will be introduced in the next section.

#### Spectrally resolved imaging with spatial resolution beyond diffraction limit

**Fig. 6.**
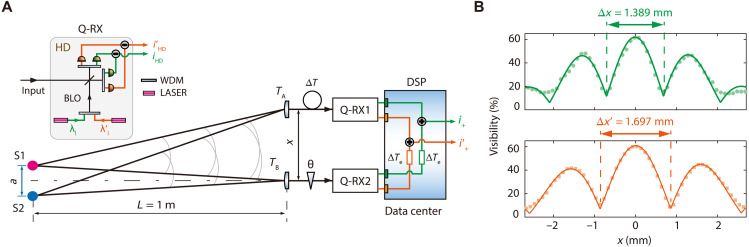
Estimation of the distance a between two independent closely spaced broadband incoherent point sources (S1 and S2). (**A**) The scheme of quantum interferometer for achieving spectrally resolvable angular resolutions beyond diffraction limit of single aperture. A pair of lenses (*T*_A_ and *T*_B_) at spatially separated locations collect the light from distant sources S1 and S2. The inset illustrates the configuration of Q-RX with BLO consisting of two CW lasers at wavelength $\lambda_{l}=$1310 nm and $\lambda_{l}^{\prime }=$ 1605 nm, respectively. Two current outputs of Q-RX1 (Q-RX2), ${\hat{i}}_{\text{HD}}\left(t\right)$ and ${\hat{i}}_{\text{HD}}^{\prime }\left(t\right)$, preserving the information of the quadrature amplitudes of input at 1310 and 1605 nm, respectively, are obtained by demultiplexing the mixing of BLO and light collected by *T*_A_ (*T*_B_). Wave superposition for light centering at $\lambda_{l}$ ($\lambda_{l}^{\prime }$) is realized by the current addition $i_{+}\left(t\right)$ [$i_{+}^{\prime }\left(t\right)$] after transferring currents of Q-RXs to data center for DSP. When $|∆T-∆T_{e}|=0$, interference can be observed, and the visibility varies with the baseline length *x* between *T*_A_ and *T*_B_. (**B**) The visibility versus *x* for the field at 1310 nm (green circles) and 1605 nm (brown squares), respectively. Green and brown curves are the fittings of data. For the individual green (brown) curve, the baseline span of two adjacent minimum is 1.389 (1.697) mm, and a=0.943±0.02 (0.944 ±0.02) mm can be calculated by Eq. 14, indicating that the spatial resolution at different wavelengths can simultaneously surpass the diffraction limit of single aperture. In the experiment, the minimum distance resolvable by individual *T*_A_ or *T*_B_ with diameter of about 0.9 mm is a≈2 mm (for λ≈1550 nm), ∆T = 5 ns, and the average photon number of the input field is ${\bar{n}}^{\prime }\eta^{\prime }\approx 0.8$*.*

We apply the quantum interferometric method in evaluating the distance between two independent point sources, which can be used to gauge the spatial resolution of an optical imaging system (*5*). As shown in Fig. 6A, *a* is the separation distance between two closely spaced incoherent sources S1 and S2, and the spectrum of each point source covers the range of 1200 to 1620 nm. A pair of fiber-coupled tiny lenses (*T*_A_ and *T*_B_), mimicking the telescopes at spatially separated locations, are used to collect light. For each lens with aperture diameter of *D* ≈ 0.9 mm, the angular resolution is $∆\phi =\frac{1.22\lambda }{D}\approx 2\times 10^{-3}$ rad for λ≈1550 nm. When the space between the point sources and collector lenses is about *L* ≈ 1 m, individual *T*_A_ (or *T*_B_) cannot distinguish S1 and S2 with distance a<LΔϕ≈2 mm for λ≈1550 nm due to the restriction of diffraction limit. By using the interferometric method, the distance a<2 mm can be measured provided that the baseline length *x* between *T*_A_ and *T*_B_ is large enough (*5*). In this quantum interferometer, the LO realized by combining multiple single-frequency lasers or by the individual lines of a laser frequency comb (*45*) helps to fully use the spectral information of broad band input and to obtain spectrally resolved complex visibility for reconstructing high spatial resolution spectral imaging over a wider frequency range. As a proof-of-principle illustration, the LO of each Q-RX is realized by combining the outputs of two CW lasers at wavelengths $\lambda_{l}=\frac{2\mathrm{\pi c}}{\omega_{l}}$ and ${\lambda_{l}}^{\prime }=\frac{2\mathrm{\pi c}}{{\omega_{l}}^{\prime }}$, respectively. For the broadband input, the frequency components $\omega_{l}\pm \Omega$ and ${\omega_{l}}^{\prime }\pm \Omega$ are selected and amplified by the bichromatic LO (BLO) of Q-RXs. The inset of Fig. 6A shows that the Q-RX is basically a HD, in which BLO is mixed with the collected light at a 50/50 BS and each output of BS propagates through wavelength division multiplexer to separate the fields at different wavelength. The two currents out of each Q-RX, ${\hat{i}}_{\mathrm{HDj}}\left(t\right)$, ${\hat{i}}_{\mathrm{HDj}}^{\prime }\left(t\right)$ (*j = 1,2*), are proportional to the amplitudes ${\hat{X}}_{j}\left(\varphi_{j}\right)$ (j = 1,2) of collected fields centering at $\lambda_{l}$ and ${\lambda_{l}}^{\prime }$, respectively.

When $|∆T-∆T_{e}|=0$, the whole spectral information of input is attainable. For the field selected by LO at wavelength $\lambda_{l}$, the power of current addition of two Q-RXs has the form

$$\langle {\hat{i}}_{+}^{2}\left(t\right)\rangle \propto g\left(x\right){|E|}^{2}\left[1+V\text{cos}\left(\theta +\varphi_{1}-\varphi_{2}\right)\right]$$ (12)

with

$$V=|\text{cos}\left(∆\beta /2\right)|=\left|\text{cos}\left(\frac{\pi \mathrm{ax}}{\lambda_{l}L}\right)\right|$$ (13)

denoting the visibility of interference, where $∆\beta =\frac{2\pi \mathrm{ax}}{\lambda_{l}L}$ is the extra phase shift for S2 relative to S1, and the function g(x) is associated with the relative intensities of light collected by *T*_A_ and *T*_B_ (see fig. S11 and the Supplementary Materials for details). Clearly, the dependence of visibility V upon the parameters of *a*, *x*, and *L* is the same as that of Michelson Stellar interferometer (*5*). When ∆β=±π, V takes the minimum value, $V_{\text{min}}.$ From the baseline lengths $x_{1}$ and $x_{2}$, which correspond to two adjacent minimum visibility, the distance

$$a=\frac{\lambda_{l}L}{|x_{1}-x_{2}|}$$ (14)

can be accurately calculated. In the sense of forming a virtual lens with the diameter of $|x_{1}-x_{2}|$, the scheme in Fig. 6A is similar to that of traditional interferometer. However, the quantum interferometer is free from the optical delay line and narrow band optical filter, which are necessary components of traditional interferometer and limit the frequency range of the collected light. Moreover, using the Q-RXs with BLO, the interference patterns at two widely separated wavelengths ($\lambda_{l}$ and ${\lambda_{l}}^{\prime }$) can be simultaneously obtained. As a result, the spectrally resolved spatial resolutions surpassing the diffraction limit can be obtained over a wide frequency range of about 40 THz.

Figure 6B plots the visibility of the interference as a function of baseline length for the light at two different wavelengths. In the experiment (see fig. S11 and the Supplementary Materials for details), individual *T*_A_ or *T*_B_ cannot resolve the distance a=0.943±0.004 mm. BLO of each Q-RX is realized by mixing the output of two lasers centering at 1310 and 1605 nm, respectively. The linewidth and power of each laser are about 100 kHz and 0.8 mW, respectively. The path imbalance induced optical delay is ∆T=5 ns. The average photon numbers of the measured input field at both 1310 and 1605 nm are ${\bar{n}}^{\prime }\eta^{\prime }\approx 0.8.$ The position of *T*_B_ placed along the optical axis (dash-dotted line in Fig. 6A) is fixed, and *x* is varied by changing the position of *T*_A_ with a step of 0.1 mm. For each setting of *x*, $|∆T-∆T_{e}|=0$, and interference is observed by scanning the relative phase θ. The visibility V is evaluated from the interference presented in the average power of current addition $\langle i_{+}^{2}\left(t\right)\rangle$ (or $\langle {{\hat{i}}_{+}^{\prime }}^{2}\left(t\right)\rangle$) (see fig. S12 for details). The data points in green circles and brown squares represent the results for $\lambda_{l}=$ 1310 nm and $\lambda_{l}^{\prime }=$ 1605 nm, respectively. The green and brown curves are the fitting of the two cases (see eq. S5). We find the baseline spans $\Delta x=|x_{1}-x_{2}|$ and $\Delta x^{\prime }=|x_{1}^{\prime }-x_{2}^{\prime }|$ read from two adjacent $V_{\text{min}}$ in green and brown curves are about 1.389 and 1.697 mm, from which a=0.943±0.017 mm and a=0.944±0.017 mm can be respectively obtained by using Eq. 12. The results agree with the prediction of Eq. 14.

#### Doppler shift induced by radial velocity down to 1 cm/s

**Fig. 7.**
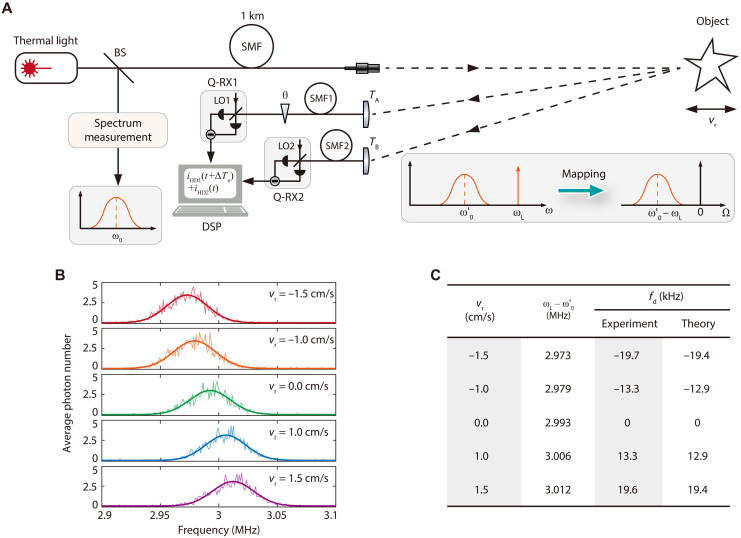
Estimating the Doppler shift induced by the radial velocity of a moving object. (**A**) The experimental scheme. The narrow band thermal light used to mimic the emission line of a star is split by a BS. The spectrum of input field centering at $\omega_{0}$ is obtained by directly measuring the light reflected by BS. A distant moving object is illuminated by the transmitted light after propagating through 1-km SMF. The spectrum of light reflected off the object is measured by the quantum interferometer, in which the amplitudes of the fields collected by lens *T*_A_ and *T*_B_ at two separated locations are respectively measured by Q-RXs (Q-RX1 and Q-RX2), and DSP technology is exploited. The center frequency of measured spectrum shifts to $\omega_{0}^{\prime }=\omega_{0}+f_{d}$ due to the radical velocity $v_{r}$-induced Doppler shift $f_{d}$. The inset illustrates the narrowband spectrum centering at $\omega_{0}^{\prime }$ is coherently mapped to RF Ω by the Q-RX with LO at $\omega_{l}$. (**B**) The spectrum extracted from the spectrally resolved interference fringes of the quantum interferometer for $v_{r}=0,\pm 1,\pm 1.5$ cm/s, clearly demonstrates that the center frequency of spectrum varies with $v_{r}$. The solid curves obtained by fitting the spectrum with a Gaussian function are used to estimate the Doppler shift. (**C**) The radical velocity-induced Doppler shift estimated from experimental measurement and theory calculation. The results indicate that the interferometer can be used to measure $v_{r}$ down to 1 cm/s with a precision of about 0.08 cm/s. In the experiment, the intensity of the input field collected by each lens (*T*_A_ and *T*_B_) is about 4 × 10^5^ photons/s, $\omega_{l}-\omega_{0}\approx 3$ MHz, $|∆T-∆T_{e}|=0$ and the frequency resolution bandwidth of DSP is 1 kHz.

We demonstrate that quantum interferometer being able to achieve spectral resolution up to $10^{11}$ can be used to measure the Doppler shift induced by the moving object with radial velocity $v_{r}$ down to 1 cm/s. The schematics is shown in Fig. 7A (see fig. S13 and the Supplementary Materials for details). The narrow band thermal input field $\hat{E}\left(t\right)=\frac{1}{\sqrt{2\pi }}\int d\omega f\left(\omega_{\text{in}}\right)\hat{a}\left(\omega \right)e^{-i\omega t}$ is used to mimic the emission line of a star, where $f\left(\omega_{\text{in}}\right)=\text{exp}\left[-\frac{{\left(\omega -\omega_{0}\right)}^{2}}{2\sigma_{0}^{2}}\right]$ with $\omega_{0}$ and $\sigma_{0},$ respectively, denoting the center frequency and width describes the spectrum of input. The input is split into two by a BS. The transmitted light illuminates the distant moving object, which is separated apart from the source by 1-km SMF. Because of the Doppler shift $f_{d}=\frac{2\omega_{0}v_{r}}{c}$ induced by the radical velocity $v_{r}$, the center frequency of the light reflected off the object is shifted to $\omega_{0}^{\prime }=\omega_{0}+f_{d}$. When the velocity of object $v_{r}$ is varied, we estimate $f_{d}$ by the quantum interferometer. The quadrature amplitudes of the light collected by lens *T*_A_ and *T*_B_ at two spatially separated locations are respectively measured by Q-RX1 and Q-RX2, and the currents of Q-RXs are processed by DSP in data center. When LO of each Q-RX is at the frequency $\omega_{l}>$ $\omega_{0}+3\sigma_{0}$, the spectrum of collected light is coherently mapped into the RF centering at $\Omega =\omega_{0}^{\prime }-\omega_{l}$ (see the inset in Fig. 7A). In the experiment, the central wavelength and bandwidth of narrow band thermal input are 1550.12 nm and ~50 kHz, respectively, $\omega_{l}-$ $\omega_{0}\approx 3$ MHz, and the intensity of light collected at each location by *T*_A_ (or *T*_B_) is about 4 × 10^5^ photons/s. When $|∆T-∆T_{e}|=0$ and FFT algorithm is applied in DSP, we achieve the interference pattern with optimum visibility for each frequency component $\omega_{l}-\Omega$, where Ω=2.9+n×∆f MHz (0≤n≤200 and ∆f=1 kHz) (see figs. S13 and S14 and the Supplementary Materials for details). According to the size of interference fringes at different frequency, we obtain the spectrum of collected light. As shown in Fig. 7B, the center frequency of the spectrum varies with the velocity $v_{r}.$ The thick solid curves (Fig. 7B) are obtained by fitting the measured spectrum with the Gaussian function, from which the center frequency of each spectrum can be accurately extracted out (Fig. 7C). Taking the spectrum of $v_{r}=0$ as a reference, which is the same as that directly measured at the reflection port of BS, the Doppler shift of the collected light is estimated to be $f_{d}=\pm 13.3$ kHz and $f_{d}=\pm 19.7$ kHz for $v_{r}=\pm 1$ cm/s and $v_{r}=\pm 1.5$ cm/s, respectively. The deviations between $f_{d}$ obtained from experiment measurement and theory calculation for different $v_{r}$ are all within the frequency-resolution bandwidth 1 kHz of DSP. The results indicate that the interferometer can be used to measure $v_{r}$ down to 1 cm/s with the precision of about 0.08 cm/s, which is two orders magnitude better than that achievable by astronomers at the current stage (*15*).

Moreover, we note that the schematics in Fig. 7A is suitable for estimating the Doppler shift of self-luminous moving object (like a star) as well. In this case, the spectrum obtained from the emission line of a stationary object serves as the reference, and the Doppler shift of moving object is related to $v_{r}$ through the relation $f_{d}=\frac{\omega_{0}v_{r}}{c}$.

## DISCUSSION

We have implemented the proof-of-principle demonstration of VLBI at optical frequency band. The quantum interferometric method for observing optical interference through direct amplitude addition by HD technique–based Q-RXs and DSP is entirely a linear process. Therefore, all the information of the complex amplitudes of input fields can be preserved even after detection. The optical signal processing functions, such as balancing paths, optical filtering, and correcting the relative phase shifting of two LOs, etc., can be performed at the electrical stage after detection. In addition to verifying the quantum effect by exploiting the input of single-photon state, we have demonstrated the VLBI can achieve ultrahigh resolution in both spatial and spectral domains. In principle, there exist no fundamental limitations to the baseline length and spectral resolution. In practice, the baseline length is mainly limited by the phase relation between the LOs at two spatially separated locations, and the spectral resolution is a trade-off between the sampling rate in DSP and time resolution. The ultrahigh spectral resolution up to 10^11^ enables the measurement of radial velocity down to 1 ± 0.08 cm/s. The orders of magnitude improvements in tolerancing the path imbalance, in spectral resolution and in accuracy of radial velocity have identified the promising future of the quantum interferometer. In our experiments, we obtain the strong LOs (LO1 and LO2) of two Q-RXs by splitting one laser output into two. However, similar to RF interferometer with independent LOs (*46*), it is worth pointing out that LO1 and LO2 do not need to be split from a common source, and transmitted to remote sites, they can be local lasers locked to synchronized optical frequency/time references for stable phase reference (*47*–*49*). With the availability of synchronizing two optical clocks separated by 920-km optical fiber (*48*), extending the baseline to hundreds or even thousands of kilometers should be in principle achievable. Moreover, when the LO of each Q-RX consists of multiple frequencies extracted from the individual lines of the laser frequency comb (*45*, *50*–*51*), the high-resolution imaging and spectroscopic capabilities will be simultaneously accessible in a single exposure. Our investigation not only opens the door for developing wide-field spatiospectral optical interferometers with baseline comparable to that of radio interferometry but also paves the way to designquantum photonic devices for information processing.

In addition, the task of measuring the second-order coherence function without bringing two optical interfering fields together can be also implemented by sharing quantum entanglement between different locations (*8*, *9*), which was first proposed Gottesman, Jennewein, and Croke (GJC). In the GJC scheme, the interference pattern was observed from two-photon coincidence measurement and the baseline length can be extended by using quantum repeater (*8*, *9*). A key feature of the GJC scheme is that the measurement for observing interference can be classified as the intensity detection. Since the two-photon events formed by the four fields from object and shared entanglement are required to be indistinguishable before the single-photon detectors (SPDs) for coincidence measurement (*8*–*10*), the optical superposition and path matching are still indispensable conditions. In contrast, the Q-Rx in our scheme measure the quadrature amplitudes of interfering field, and the optical interference is recovered after detection and digitally processing the photocurrents of Q-Rxs. Hence, the optical superposition and path matching are no longer required before detection. Moreover, unlike the quantum network for long-distance quantum state distribution and manipulation, which is currently in the infancy stage, the technologies for supporting the amplitude measurement based VLBI, including the laser technology, HD, DSP, and optical frequency metrology, are mature and ready to use.

## MATERIALS AND METHODS

### Experimental setup

The experimental setup of scheme in Fig. 1 is shown in detail in fig. S1. The pulsed input of heralded single photons centering at 1553.3 nm is in nearly single mode. The 50/50 BS splits the single-photon state input into two independently propagating fields. Instead of bringing the two optical fields to a common location, the interference is formed by the coherent addition of the photocurrents of two Q-RXs, which are basically HDs and measure the amplitudes of two fields at different locations and at different times, respectively. The photocurrents of Q-RXs converted into voltages are sent into a DSO triggered by the heralding signal of SPS for DSP. We record and process the voltage signals in time domain when the relative phase between two distinguishable paths is scanned at a rate of about 2 to 3 Hz. Interference is observable when the adjustable electrical delay $∆T_{e}$ is properly set to balance the optical delay ∆T originated from the difference between two separate optical paths.

In the experiment, the pulsed pump of the heralded SPS and LO, respectively, centering at 1549.3 and 1553.3 nm are created by passing the output of a mode locked fiber laser through a dual-band filter (F1) (*40*, *41*). The central wavelength and FWHM of the laser are about 1550 and 60 nm, respectively. Hence, the LO is synchronized with SPS. Moreover, to ensure the response time of our high-efficiency Q-RX (*T*_R_ ≈ 50 ns) is fast enough to resolve the single-photon events between two adjacent pulses, the repetition rate of the laser is reduced to 5.3 MHz, which is realized by chopping the laser output with an EOM (electro-optic modulator)–based optical switch so that the repetition rate of laser is decreased by seven times. The isolation of the chopped laser output is greater than 10 dB, and the influence of small residual pulses is negligibly small. The LOs of Q-RX1 and Q-RX2, labeled as LO1 and LO2, are obtained by splitting the LO with a 50/50 BS. The relative phase drifting between two pulse trains of LO1 and LO2 is managed to be negligibly small within a few seconds. The total detection efficiency [including the heralding efficiency of SPS (∼50%), mode-matching between LO and single-photon state (∼45%) (see the Supplementary Materials for details), the transmission efficiency induced by other optical components in system (∼75%), and detection efficiency of Q-RX (∼95%)] is ∼16%.

When the input of single photons is replaced with pulsed thermal state (see the Supplementary Materials for details), the trigger of DSO in fig. S1 is removed, and the wavelengths of pass-band filters (F1 and F2) are simultaneously adjusted so that the spectra of thermal field and LO centering at 1560 nm are still the same. Under this condition, the mode matching between the pulsed thermal field input and LO of each Q-RX is about 45%. Moreover, to increase the data rate, the Q-RXs are replaced with the ones having quantum efficiency and response time of about 70% and 10 ns, respectively. In the meantime, the EOM-based switch for decreasing the repetition rate of mode locked laser is removed.

### Generation of single-photon state in nearly single mode

As show in fig. S1, the heralded single-photon state is generated from the χ^(3)^ nonlinearity in 300-m-long dispersion-shifted fiber (DSF) through the pulse-pumped spontaneous four-wave mixing (*40*, *41*). The DSF is cooled to 2.1 K by a cryostat to suppress the background noise contributed by spontaneous Raman scattering. The detection events of the SPD placed in idler channel are used to herald the presence of single photons in the signal channel. The central wavelengths of pulsed pump, heralded single photon, and heralding idler fields are 1549.3, 1553.3, and 1545.3 nm, respectively, and the FWHM of the three fields are 0.6, 1.1 and 0.6 nm, respectively. We characterize the SPS by measuring the heralding efficiency $\eta_{h}$, the photon statistics $g^{\left(2\right)}$, and mode number $M_{s}$ (see fig. S2 and the Supplementary Materials for details). The results show that we have $\eta_{h}=50\%$, $g^{\left(2\right)}=0.07\pm 0.006$, and $M_{s}\leq 1.3$ when detection rate of heralding idler field is about 4.85 × 10^4^ Hz. In addition, we calibrate the SPS by using HD with strong LO to measure its amplitude probability histogram (see fig. S3 and the Supplementary Materials for details). We find the variance of the histogram for our SPS is about 1.2 dB higher than that for vacuum state ∣0⟩, showing that the amplitude of the heralded single-photon state ∣1⟩ can be effectively measured.

### VLBI with optical path imbalance over 10^6^ larger than coherence length

In this experiment, the thermal field input is in nearly single mode (see the Supplementary Materials for details), and the repetition rate of pulsed thermal light is 50 MHz. The response time $T_{R}$ and quantum efficiency of each Q-RX are about 10 ns and 70%, respectively. Considering the mode-matching efficiency between the pulsed thermal field and LO, the total detection efficiency is around 30%.

Figure S4 shows a typical set of data and results for recovering interference. In this measurement, the average photon number of the field incident on each Q-RX is ${\bar{n}}^{\prime }\approx 27$ photons per pulse, and the relative phase between two optical paths is varied by sweeping the voltage on the PZT-FS at a rate of several hundred hertz. It takes several steps to process data acquired by the DSO. First, the output of each Q-RX is recorded at a sampling rate of 10 GHz. The time window is chosen to be 5 ms, and the results of 2.5 × 10^5^ optical pulses are analyzed. Figure S4 (A and B, respectively) plots the raw data sampled from the current of individual Q-RX and the current addition of two Q-RXs in the time window of 500 ns, showing that the response time of each Q-RX is fast enough to resolve two adjacent optical pulses. Moreover, the profile of each electrical pulse appears to be a dip followed by a peak. The appearance of dip is due to imperfect electronic balance between two photodiodes of Q-RX, while the peak carries the information of quadrature amplitude of light. We then extract the peak of each pulse and correct it by subtracting the mean value for all the peaks of the 2.5 × 10^5^ pulses. As shown in fig. S4C, the quadrature amplitude measured by individual Q-RX can be both positive and negative, and no interference is observable. Using the same procedure to process the data, we obtain the current addition $i_{+}\left(t\right)=\langle {\hat{i}}_{+}\left(t\right)\rangle$ shown in fig. S4D, from which we can observe the interference pattern. Figure S4E plots the average of current power $\langle {\hat{i}}_{+}^{2}\left(t\right)\rangle$ (with average time of 6 μs or 300 pulses), from which the visibility of interference can be conveniently evaluated. For the sake of clarity, the average current power of individual Q-RX $\langle {{\hat{i}}_{\mathrm{HDi}}}^{2}\left(t\right)\rangle \;\left(i=1,2\right)$ (represented by the blue and green traces) are also plotted. We find that the visibility of interference (pink trace) is about 94%, whereas no interference pattern is observable from the current power (blue and green traces) of individual Q-RX. The absence of interference indicates that there is no coherence between the input field and the LO. The role of two LOs in Q-RXs is to form HD for measuring the amplitudes of thermal fields at different locations.

### CW broadband thermal light source

The CW broadband thermal light input in Fig. 4 is obtained by using BSs to combine three kinds of sources, which are fiber-coupled light-emitting diode and the spontaneous emission of erbium-doped fiber amplifiers in C and C + L bands, respectively. Figure S5 illustrates that the spectrum of the source covers the wavelength range of 1200 to 1600 nm.

### The SNL and linear dynamic range of Q-RX

In our measurements, the SNL is 10 dB higher than the electronic noise power of Q-RX. For clarity, all the results in Fig. 4 (B to G) are normalized to their corresponding SNL. The levels of SNL are ${\langle {\hat{i}}_{+}^{2}\rangle }_{\mathrm{SNL}}=9.3\times 10^{-5}$ V^2^ in Fig. 4B, ${\langle {\hat{i}}_{+}^{2}\rangle }_{\mathrm{SNL}}$ = 1.6 × 10^−7^ V^2^ in Fig. 4 (C and D), ${\langle {\hat{i}}_{+}^{2}\rangle }_{\mathrm{SNL}}=2.4\times 10^{-6}$ V^2^ in Fig. 4E, and ${\langle {\hat{i}}_{+}^{2}\rangle }_{\mathrm{SNL}}$ = 6.6 × 10^−10^ V^2^ in Fig. 4 (F and G).

On the other hand, for the thermal field input, the variance of amplitude measured by each Q-RX is $\langle {\hat{X}}^{2}\rangle =2\eta^{\prime }{\bar{n}}^{\prime }+1$, where ${\bar{n}}^{\prime }$ is the average photon number per mode, and $\eta^{\prime }$ is the detection efficiency. To evaluate the linear dynamic range of Q-RX, we need to assess the average photon number ${\bar{n}}^{\prime }$ at first. Different from the case of input in single temporal mode (Fig. 3), it is not straightforward to estimate the average photon number ${\bar{n}}^{\prime }$(per hertz) of broadband CW thermal field. The detailed procedure and results of characterizing the SNL and linear dynamic range of Q-RXs for CW broadband input are described in the Supplementary Materials (see fig. S6 for detail).

### VLBI with broadband input and ultrahigh spectral resolution

The plots on the left side of fig. S7 show a typical set of data for recovering interference when the input is CW broadband thermal field. In the measurement, $|∆T-\Delta T_{e}|=0$, and the sampling rate is set at 1.25 GHz. In the time window of 50 ms, there are 6.25 × 10^7^ data points. Figure S7A plots the raw data sampled from the current of individual Q-RX, showing that the current of individual Q-RXi $i\left(t\right)=\langle {\hat{i}}_{\mathrm{HDi}}\left(t\right)\rangle$ (i=1,2) can be both positive and negative, and no interference can be observed because the thermal field input has no phase correlation with the LO field. An interference pattern is observable in the current addition of two Q-RXs $i_{+}\left(t\right)=\langle {\hat{i}}_{+}\left(t\right)\rangle$ (fig. S7B). To compare the results with SNL, we also record the current addition ${\langle {\hat{i}}_{+}\left(t\right)\rangle }_{\mathrm{SNL}}$ by blocking the input of each Q-RX, as shown in fig. S7C. To better observe the interference fringe and evaluate the visibility, we further calculate average power of the currents in fig. S7 (B and C) and normalize the results to SNL when the average time is chosen to be 40 μs, as shown in Fig. 4B.

The plots on the right side of fig. S7 demonstrate two kinds of interference fringes. Figure S7D shows a typical fringe pattern in Fig. 4C, which corresponds to the field at the individual frequency $\omega_{l}+n\times$ 200 kHz, where n is an integer in the range of 0≤n≤500. The fringe in fig. S7E is obtained by multiplexing nine traces in Fig. 4C. Clearly, the noise of the fringe in fig. S7E is much smaller than that in fig. S7D (see the Supplementary Materials for details).

Figure S8 shows the results when $|∆T_{e}-∆T|$ = 5 μs $\gg T_{R}$ and the sampling parameters of DSP are the same as those in fig. S7. In this case, the interference in time domain is unobservable from the raw data sampled from current addition of two Q-RXs $i_{+}\left(t\right)=\langle {\hat{i}}_{+}\left(t\right)\rangle$ (inset in fig. S8A) and from the average power of current addition $\langle {\hat{i}}_{+}^{2}\left(t\right)\rangle$ (fig. S8A). The interference in frequency domain is unobservable from the normalized power spectrum of current addition S(Ω) (pink trace in fig. S8B) as well. This is because the RBW of DSP applied in analyzing S(Ω) is 200 kHz, which is limited by the sampling rate and memory size. In this case, the RBW is too large to resolve the variation period ~100 kHz of the S(Ω) (see Eq. 11 and Fig. 4G).

The periodically varied visibility in Fig. 4 (F and G) is obtained when the power spectrum S(Ω) is analyzed by passing the output of each Q-RX through a 2-MHz low-pass filter and selecting a smaller spectral range (see fig. S8B for details) with the central frequency and bandwidth of 1.9 MHz and 200 kHz, respectively. Under this condition, sampling rate, sampling time, and the RBW in DSP are 10 MHz, 5 s, and 0.8 kHz, respectively.

When $|∆T-\Delta T_{e}|=0$, the visibility of interference pattern extracted from S(Ω) can reach the maximum for the field $\hat{E}\left(t\right)=E_{+}e^{-i\left(\omega_{l}+\Omega \right)t}+E_{-}e^{-i\left(\omega_{l}-\Omega \right)t}$ with $E_{+}$ and $E_{-}$ independent of each other. In the process of recovering the interference patterns for each frequency component $\omega_{l}\pm \Omega$, the setting of RBW does not influence the observation of interference when the bandwidth of input field is much broader than the frequency bandwidth of Q-RX. Note that in the process of plotting Fig. 4D, the influence of electronic noise of Q-RX at different frequency Ω (see fig. S8B for details) is taken into account.

## Supplementary Materials

### This PDF file includes:

Supplementary Text Figs. S1 to S14 References
