# Supplementary material for: Quantum interference of single photons without optical superposition: Toward high resolution imaging in spatial and spectral domains
Source: Sci Adv. 2026 Apr 22;12(17):eaea9701. doi: 10.1126/sciadv.aea9701 (PMC13101880; doi:10.1126/sciadv.aea9701)
Supplement: Supplementary file 1 — Supplementary Text Figs. S1 to S14 References [file sciadv.aea9701_sm.pdf]

Science Advances
AAAS

Supplementary Materials for

Quantum interference of single photons without optical superposition: Toward high resolution imaging in spatial and spectral domains

Yunxiao Zhang *et al.*

Corresponding author: Xiaoying Li, xiaoyingli@tju.edu.cn; Z. Y. Ou, jeffou@cityu.edu.hk

Sci. Adv. **12**, eaea9701 (2026)
DOI: 10.1126/sciadv.aea9701

This PDF file includes:

Supplementary Text
Figs. S1 to S14
References

Supplementary Text

1. Characterization of the heralded single photon sources

We first measure the heralding efficiency, photon statistics and mode number of the heralded single photon source by using photon counting technic (*40,41*). Heralding efficiency η_h is measured by using two fiber-coupled superconducting nanowire single photon detectors to detect the signal and idler photons. The detection efficiency of each SPD is about 80% and its dark counts is less than 500 counts per second. The counting rate of SPD (R_h) placed in heralding idler channel increases with pump power. Figure S2(A) plots the coincidence of two SPDs as a function of the counting rate in heralding idler channel. The data points well fit a solid line, showing the ratio between the coincidence rate C_c and heralding rate R_h is about 0.4. Taking the detection efficiency of SPD (80%) in heralded single photon channel into account, we have $\eta_h \approx$ 50%. Figure S2(B) shows the intensity correlation function of SPS $g^{(2)}$ versus the heralding rate R_h, which is measured by sending the single photon state through HBT interferometer consisting of a 50/50 beam splitter and two SPDs (*52*) (not shown in fig. S1). The value of $g^{(2)}$ increases with heralding rate due to the influence of multi-photon events. But even if the heralding rate is up to 100 KHz, the value $g^{(2)} = 0.14 \pm 0.006$ is still well below the classical Poissonian light limit of 1. In particular, we have $g^{(2)} = 0.07 \pm 0.006$ when the counting rate in heralding idler channel is about 4.85×10^4 Hz, at which the experimental results in Fig. 2 are obtained. Moreover, considering the thermal nature of individual idler field, we can estimate the mode number of heralding idler field $M_h \approx 1.3$ from the relation $g_h^{(2)} = 1 + \frac{1}{M_h}$ by sending the individual heralding field into the HBT interferometer and measuring its intensity correlation function $g_h^{(2)} = 1.75$. Since the mode number of heralding field sets the lower bound of the modal purity of SPS, its straight forward to deduce of mode number of SPS, $M_s \leq 1.3$.

We then calibrate the source of single photons by using homodyne detector (HD) to measure the quadrature amplitude $\hat{X} = \hat{a}e^{-i\varphi} + \hat{a}^{\dagger}e^{i\varphi}$, where φ is the phase of the strong LO. A single photon state $|1\rangle$ is inherently non-Gaussian and its amplitude probability distribution is irrelevant to φ, so the calibration result is insensitive to the LO phase. Ideally, the measured result obeys probability distribution of $P_1(x) = (2x^2/\pi)\, exp(-x^2)$ (*43*), where x refers to the operator $\hat{X}$ measured by HD. In practice, there inevitably exists the contribution of vacuum induced by non-ideal heralding efficiency and non-ideal HD detection. For the overall detection efficiency η, the amplitude probability distribution can be modeled by

$$P(x) = \eta P_1(x) + (1-\eta)P_0(x), \tag{S1}$$

where $P_0(x) = (2/\pi)\, exp(-x^2)$ denotes the amplitude probability distribution for vacuum state $|0\rangle$.

In the process of measuring $P(x)$, the BS and SMF placed in front of Q-RX2 in fig. S1 are removed so that the heralded single photon source is directly measured by one Q-RX, which is basically a HD. The HD consists of two identical photodiodes. Before measuring the quadrature amplitude of SPS, we first evaluate the response function of HD ($k(t)$) by analyzing the current pulses out of individual photodiode. The inset in fig. S3(A) plots the shape of one electrical pulse of individual photodiode, showing the raising edge of $k(t)$ is sharp and the FWHM is about 50 ns, but the falling edge will last to about 150 ns. To effectively evaluate the quadrature amplitude of each pulse, we record the current pulse in time domain and extract its maximum value within the timing window of around 50 ns (marked by the gray shadow area in fig. S3).

When the quadrature amplitude of single photon state is measured by HD, the LO power is about 1.12×10^9 photons/pulse. To match the mode of SPS, the central wavelength and FWHM of LO are 1553.3 and 1.1 nm, respectively. The output of HD is converted into voltage and sent to a digital storage oscilloscope trigged by the heralding signal of the single photons. Figure S3(A) plots a typical trace of one electronical pulse out of HD, in which the height of the peak is proportional to the value of quadrature amplitude of input field. For each set of data, 1.75×10^4 pulses are recorded and processed. We extract the peak of each pulse and correct it by subtracting the mean value for all the peaks of the recorded electronic current pulses. Figure S3(B) plots the value of extracted peaks when the input of HD is heralded single photon state $|1\rangle$ (blue) and vacuum state $|0\rangle$ (orange), respectively. By performing probability histogram analysis to these peak values (the dots in fig. S3(C)), we find the variance of the histogram for SPS (blue) is about 1.2 dB higher than that for vacuum state (orange). Fitting the histogram of single photon state $|1\rangle$ in fig. S3 (C) with eq. (S1), we find the overall detection efficiency is $\eta = 19.2\%$. Considering heralding efficiency of SPS ($\eta_h \approx 50\%$), the quantum efficiency of the HD system ($\eta_{HD} = 95.0\%$) and non-ideal the transmission efficiency of (90%), mode-matching efficiency between LO and single photon state is deduced to be $\sim 45\%$. Finally, we repeat the measurement by changing the pump power of SPS to vary the trigger rate. As shown in fig. S3(D), for the heralding rate R_h in the range of 30-120 kHz, the variances of the histogram of SPS (blue squares) are about 1.2 dB higher than that for vacuum state (orange triangle).

2. Generation of thermal state in nearly single temporal mode

The pulsed thermal field input is simply obtained by removing the trigger of heralding idler field since the individual signal or idler field generated by spontaneous four wave mixing is in thermal state (*40*, *44*). When the wavelength of individual signal field is close to the gain peak of spontaneous four wave mixing, the intensity (mode number) of the thermal field in signal band increases (decreases) with the increase of pump power (*44*). In the process of generating pulsed thermal state, the DSF and the central wavelength of pump pulses are the same as those in fig. S1, but the laser system is replaced by the one having repetition rate of 50 MHz. Moreover, the filter F2 in fig.S1 is changed so that the central wavelength of individual signal thermal field is changed to 1560 nm, which is close to the gain peak of spontaneous four wave mixing. When the pump power is 6×10^8 photons/pulse, the average number of individual signal thermal field is up to 60 photons/pulse, and the mode number deduced from the measured intensity correlation is $M_{th} \approx 1.4$. The average photon number of thermal field input can be adjusted by placing an adjustable attenuator in front of the BS (not shown in fig. S1).

3. Details for characterizing the linear dynamic range of quantum receivers

In this experiment, the input is obtained by passing the spontaneous emission of EDFA through a polarization beam splitter and a fiber coupled narrow band filter. In this case, the CW input field is written as $\hat{E}(t) = \frac{1}{\sqrt{2\pi}} \int d\omega f(\omega_{in}) \hat{a}(\omega) e^{-i\omega t}$, where $f(\omega_{in}) = \exp\left[-\frac{(\omega-\omega_{in})^2}{2\sigma_{in}^2}\right]$ describes the spectrum of filter. The central wavelength and FWHM of input field are $\lambda_{in} = \frac{2\pi c}{\omega_{in}}$ =1550.1 nm and $\Delta B_{in} = 2.35\sigma_{in}$ ≈300 MHz, respectively. We estimate the average photon number per Hz through the relation: $\bar{n}' = \frac{I_{in}}{h\nu\Delta B_{in}}$, where I_{in} is the power of input and $h\nu$ is the energy of individual photon. During the measurement, the average photon number $\eta'\bar{n}'$ is adjusted by using the combination of a power meter and calibrated attenuators, the wavelength of LO is 1550.1 nm, and the power of LO is significantly higher than that of input ($|\mathcal{E}|^2 \gg I_{in}$). When the bandwidth ΔB determined by the response function of Q-RX and the electrical filter applied in

DSP is much smaller than the bandwidth of input field, we have $\hat{E}(t) = \frac{1}{\sqrt{2\pi}} \int d\omega \hat{a}(\omega) e^{-i\omega t}$, and the current power of individual Q-RX is expressed as

$$\langle \hat{\imath}_{HD}{}^2(t) \rangle = \langle i^2 \rangle_{SNL} (2\eta' \bar{n}' + 1) \qquad \text{(S2)}$$

with

$$\langle i^2 \rangle_{SNL} \propto |\mathcal{E}|^2 \Delta B \qquad \text{(S3)}$$

denoting the shot noise level (SNL).

Figure S6(A) plots the average power of SNL $\langle i^2 \rangle_{SNL}$ (in terms of voltage) versus the power of LO when the bandwidth of electrical filter applied in DSP is 100 MHz and the input of Q-RX is blocked. Fig. S6(A) shows that the electronic noise power of Q-RX is 10 dB lower than the SNL when the power of LO is about 0.8 mW. Figure S6(B) demonstrates the normalized average of current power of Q-RX ($\langle \hat{\imath}_{HD}{}^2(t) \rangle$) when $\eta' \bar{n}'$ is changed from 0.05 to 1×10^5. We fit the data (red dots) with eq. (S2) when the measurement results are normalized to the shot noise level $\langle i^2 \rangle_{SNL} = 2.64 \times 10^{-5} V^2$. The data well agrees with the linear fitting (blue curve) when $\eta' \bar{n}'$ is in the range of 0.05 to 1.6$\times 10^4$. When $\eta' \bar{n}'$ is greater than 1.6×10^4, the data deviates from the linear fitting due to the saturation effect. The result indicates that the linear dynamic range of the Q-RX is about 54 dB. Moreover, we repeat the measurement by varying bandwidth of electronic filter in DSP. During the measurement, the power of LO is fixed at about 0.8 mW. The measurement results show that the relation between the shot noise level $\langle i^2 \rangle_{SNL}$ and ΔB agree with the prediction of eq. (S3) when ΔB <100 MHz, and the linear dynamic range characterized by the normalized average power of Q-RX does not vary with ΔB if ΔB is much smaller than the bandwidth of input field.

The average photon number of broadband input can be derived from the size of interference fringe as well. When $|\Delta T - \Delta T_e| = 0$, the interference with maximum visibility $V = \frac{\langle i_+{}^2 \rangle_{max} - \langle i_+{}^2 \rangle_{min}}{\langle i_+{}^2 \rangle_{max} + \langle i_+{}^2 \rangle_{min}}$ can be recovered from the average power of current addition $\langle \hat{\imath}_+^2(t) \rangle$, where the maximum and minimum values $\langle i_+{}^2 \rangle_{max}$ and $\langle i_+{}^2 \rangle_{min}$ correspond to the peak and valley of the fringe, respectively. Accordingly, the average photon number $\eta' \bar{n}'$ of the field measured by the interferometer can be extracted from the relation

$$\frac{\langle i_+{}^2 \rangle_{max}}{\langle i_+{}^2 \rangle_{min}} = 4\eta' \bar{n}' + 1. \qquad \text{(S4)}$$

Above experimental results and analysis show that the SNL of Q-RX varies with the power of LO and bandwidth of electrical filter applied in DSP. For clarity, the average powers of current additions $\langle \hat{\imath}_+^2(t) \rangle$ measured in our experiments are normalized to SNL.

4. Multiplexing the spectrally resolved interference patterns for noise reduction

For the spectrally resolved interference $S(\Omega)$ in Fig. 4C, the interference pattern for each frequency component $\omega_l \pm \Omega$ with $\Omega = n \times \Delta f$ MHz ($0 \le n \le 500$ and $\Delta f = 200$ kHz) inevitably carries noise N_i and can be expressed as $S_i(t) = Sig_i + N_i$. Under the condition of broadband input and $|\Delta T - \Delta T_e| = 0$, we can assume the signal size (Sig_i) and noise power ($N_i{}^2$) are independent of the frequencies. For the Q-Rxs with bandwidth ΔB, the maximum number of interference fringes extracted from Fig. 4C is $n_{max} = \frac{\Delta B}{\Delta f}$. After multiplexing the interference fringes at different frequencies, the interference fringe can be written as $S_m(t) = Sig_i + N_i/\sqrt{n_{max}}$, which illustrates that the noise of interference fringe is reduced by a factor of $\sqrt{n_{max}}$.

5. Experimental details on measuring the spectrum of an absorption medium

A micro-ring resonator (MRR) side-coupled to a bus waveguide is exploited for observing the polarization dependent absorption (*53, 54*). As shown in fig. S9(A), the light is coupled from input waveguide into the ring structure via evanescent field coupling. The high Q cavity is in resonance when the wavelength of the light fits a whole number of times inside the optical length of the ring: $\lambda_{m=}\frac{2\pi R n_m}{m}$ $(m = 1,2,3 ...)$, where λ_m is the wavelength of the mth resonance with effective index n_m, R is the radius. The cross-section geometry of the MRR with $R = 135$ µm is 2 µm ×1µm, so the values of n_m in transverse electric (TE) and transverse magnetic (TM) modes are slightly different due to the influence of waveguide dispersion. As a result, the m-order resonance wavelengths in TE and TM polarization modes are different. When the broadband CW thermal light (see fig. S5) is launched to the input port of MRR, the transmission spectrum measured at the pass port will show dips around the ring resonances. Figure S9(B) demonstrates a part of the whole spectrum in TE and TM polarization modes. The resonance induced absorption dips with wavelength interval of about 1.6 nm are observable in both TE and TM modes. But the detailed structure of these dips cannot be obtained from the results measured by optical spectrum analyzer (OSA) because of its limited wavelength resolution of 0.02 nm.

We measure the spectrum in the vicinity of the dip around 1550.12 nm by inserting the MRR in the new interferometer (Fig. 4A). We recover optical interference fringes from the power of current addition $\langle \hat{\imath}_+^2(t)\rangle$ for LO at different wavelengths. The maximum (red dots) and minimum (green dots) in fig. S10(A) are extracted from the size of interference patterns obtained by changing wavelength of LO from 1549.9 nm to 1550.4 nm with the tunning step of 0.01 nm. Note that the minimum values $\langle \hat{\imath}_+^2\rangle_{min}$ of interference fringes always overlap with the SNL ($\langle \hat{\imath}_+^2\rangle_{SNL} = 8.9 \times 10^{-6} V^2$). According to eq. (S4), the spectrum of absorption line, represented by the average photon number versus the wavelength of input, is derived. As shown in fig. S10(B), the intensity asymmetry in the two sides of the dip is observable, but the structure of the dip is unidentifiable due to the coarse tunning step of LO. By tunning the LO with a smaller step of 3×10^{-4}nm, the detailed structure of the dip can be obtained (Figs. 5B-5D in the main text).

To verify the validity of the spectrum measured by new interferometric method, we also perform measurement by replacing the broadband thermal light source in fig. S9 with a frequency tunable laser. In this case, we measure the power versus wavelength at the pass port and find the FWHM of depth of the dip (around 1550.1 nm in TM mode) are about 300 MHz and 95%, respectively, which are consistent with of the results in Fig. 5D.

6. Details of resolving two independent incoherent point sources by the new interferometric method

Figure S11 shows the experimental setup of resolving two independent incoherent point sources by using the new interferometric method. The point sources S1 and S2 with short distance *a* between them emit continuous wave broadband thermal light. The fiber coupled lenses (T_A and T_B) used to imitate the function of telescopes in optical stellar interferometer are 1 m away from S1 and S2. The diameter of the clear aperture of both T_A and T_B is about 0.9 mm.

The spectrum of each broadband CW light source in fig. S11 is similar to that in fig. S5. To ensure the intensity of light collected by each lens (T_A, T_B) is not too weak to be observed, the output of each fiber coupled thermal light source is reshaped into a Gaussian beam by a grin lens (GL). The beam waist with diameter of about 0.18 mm is about 10 mm away from grin lens, and

the diameter of the Gaussian beam spreads to ~2.5 mm when the propagation distance is ~1 m (see the inset in fig. S11). To have two sources very close to each other, a 50/50 beam splitter (BS) is used to reflect and transmit the light out of two GLs, respectively. The beam waists of both reflected and transmitted light are located at the BS (labeled as S1 and S2), and the short distance between them can be flexibly changed.

The lens T_B with equal distance between the point sources S1 and S2 is placed along the optical axis represented by dash-dotted line. In principle, we can use the fiber coupled lens T_A to collect light with propagation direction the same as that of T_B, and the distance x between T_A and T_B in the vertical direction of the optical axis is the baseline length of the interferometer. In order to flexibly change the baseline length, we place a 50/50 beam splitter (BS2) in front of T_B, and use another fiber couple lens T_A' to collect the reflected light. T_A' is the mirror image of T_A with respect to BS2. The baseline length x can be automatically adjusted by fixing T_B and moving the position of T_A' mounted on a motorized translation stage.

The inset in fig. S11 shows the intensity of light collected by individual lens T_A' at different position in two cases. In the measurement, the distance between S1 and S2 is $a\approx1.5$ mm, and the position of T_A' is changed by moving the motorized translation stage. When only one source S1 (S2) is turned on, the results represented by red circles (blue stars) illustrate the intensity distribution follows Gaussian distribution with FWHM of about 2.5 mm. When both S1 and S2 are turned on, the intensity (black squares) is approximately in Gaussian distribution with FWHM of about 3.2 mm. For the light collect by T_A' at different location, and the intensities contributed by the sources S1 and S2 are different unless x=0.

According to the interference formed by measuring the amplitude of light collected by T_A' and T_B with Q-RX1 and Q-RX2 and digitally processing the photocurrents of two Q-RXs, we can estimate the distance between S1 and S2 with resolution beyond the limit of individual lens. Figure S12 shows a typical set of interference pattern for the input selected by the local oscillator $\lambda_l' = 1605$ nm. During the measurement, the average photon number for input of each Q-RX is $\eta'\bar{n}' \approx 1$, T_A' is a mirror image of T_B, i.e, the baseline length is x=0. When the phase θ between two optical paths is scanned, the current of each Q-RX is sampled and stored by the digital storage oscillator with a sampling rate of 500MHz, and the condition $\Delta T_e = \Delta T = 0$ holds. The procedure in analyzing the currents of two Q-RXs is similar to that in obtaining fig. S7 and Fig. 4B. The results in fig. S12 include the average power of the current of individual Q-RX $\langle \hat{\imath}_{HDi}{}^2(t)\rangle$ (i=1,2) (green dots) and that of current addition of two Q-RXs $\langle \hat{\imath}_+^2(t)\rangle$ (blue dots). Each data point in fig. S12 is obtained by calculating the average in every $40\ \mu s$ and normalizing the average power to the SNL (orange dots) $\langle \hat{\imath}_+^2\rangle_{SNL} = 5.68 \times 10^{-5}$ V^2. The average current power of individual Q-RX (green dots) stays constant, illustrating that S1 and S2 are independent sources. The blue curve is the fitting of the normalized average power of current addition (blue dots), indicating the visibility is about 67%, which agrees with the theoretically expected maximum visibility $V_t = \frac{2\eta'\bar{n}'}{2\eta\bar{n}'+1}$ with $\eta'\bar{n}' \approx 1$.

To evaluate the distance a between S1 and S2, we measure the visibility of the interference when the baseline length x is changed by varying the position of T_A' mounted on a transition stage. The visibility varies with x, as shown in Fig. 6B in the main text. Without loss of generality, we take the data obtained by LO at wavelength λ_l as an example. We fit the data represented by green circles by the equation

$$V \propto g(x)[1 + \gamma\cos(\Delta\beta/2)] + C. \qquad \text{(S5)}$$

where $\Delta\beta = \frac{2\pi a x}{\lambda L}$, and $g(x) = Ae^{-(\frac{x-d}{\sigma})^2}$ describes the intensity distribution of light collected by T$_A$′. Comparing with Eqs. (12) and (13) in the main text, there are extra terms γ and C in eq. (S5). This is because the contribution of S1 and S2 to the light collected by T$_A$′ is not the same for $x \neq 0$. Even if the relation $\Delta\beta = \pm\pi$ is satisfied, complete destructive interferometer with $V_{min} = 0$ cannot be obtained. Fortunately, the formula used to deduce short distance $a = \frac{\lambda L}{|x_1 - x_2|}$ is irrelevant to γ and C, where x_1, x_2 denote the values of baseline length corresponding to two adjacent minimum visibility.

In addition, we note that the results in Fig. 6 are simply analyzed in time domain only. If the minimum frequency resolution Δf is reduced by enlarging the sampling time and n_{max} sets of interference fringes at the frequency $\omega_l \pm \Omega$ with $\Omega = n \times \Delta f$ MHz are simultaneously extracted out by applying electronic filters in digital signal processing (similar to the results in Figs. 4 B-4G), the signal to noise ratio (SNR) of the measurement can be increase by a factor of $\sqrt{n_{max}}$ after multiplexing the information read from each fringe pattern. As a results, the SNR with the same level as that of the single interference pattern for $\bar{n}$ can be obtain for $\bar{n}' = \frac{\bar{n}}{\sqrt{n_{max}}}$. Moreover, if number of Q-Rx is increased from 2 to N to form an array (*3,7*), we then get the space to further reduce the average photon number to $\bar{n}' = \frac{\bar{n}}{\sqrt{N n_{max}}}$. For example, when the response bandwidth of detector, resolution unit and number of Q-Rxs are ΔB =10 GHz, $\Delta f = 1$ kHz, and N=10, respectively, we have $n_{max} = 10^7$. In this case, the SNR read from single interference fringe for $\bar{n} \approx 1$ is the same as that obtained by multiplexing n_{max} set fringe patterns and by reducing the average photon number of each fringe pattern to $\bar{n}' \approx 10^{-4}$, at which the stellar photon (with bandwidth 10 GHz) rate reaching each receiving station is about 10^6 photon per second.

7. Details of measuring the Doppler shift induced by radial velocity down to 1 cm/s

The experimental setup of scheme in Fig. 7A is shown in detail in fig. S13 (A). The narrow band thermal input field used to mimic the emission line of a star is the scattered light of a coherent field from a single frequency laser by a rotating ground glass disk. A mirror mounted on motorized translation stage is used to mimic a moving object with different radial velocity v_r. The light reflected off the mirror is then coupled back into the 1 km long single mode fiber (SMF) and directed to the new type of interferometer by a circulator (Cir). In the interferometer, the reflected light goes through a 50/50 beam splitter (BS2), and two split fields independently propagate to spatially separated locations (A, B). At each location, the quadrature amplitudes of the reflected light are respectively measured by Q-RX1 and Q-RX2. The strong LO1 and LO2 are obtained from a single frequency laser at ω_l. The difference between ω_l and the center frequency of input (ω_0) is about 3 MHz, which is about 60 times larger than the FWHM (~50 kHz) of input field. The currents of two Q-RXs are sent into a digital storage oscilloscope for DSP, by which the spectrum of reflected light can be achieved after analyzing the photocurrent addition $i_+(t) = \langle \hat{\imath}_+(t) \rangle$ in frequency domain (around $\Omega = (\omega_l - \omega_0) \pm 100$ kHz) under the condition of $\Delta T_e - \Delta$T=0.

Figure S13(B) shows the normalized power spectrum $S(\Omega)$ for the radial velocity v_r=0, ± 1, ± 1.5 cm/s. During the experiment, a 20 MHz low-passing filter is applied to the current addition of two Q-RXs. The sampling rate and sampling time of DSP is 100 MHz and 1 s, respectively. To measure the Doppler shift induced by the radial velocity down to level 1 cm/s, we apply the algorithm of Fast Fourier Transform (FFT) and analyzing the spectrum in the range

2.9-3.1 MHz with a resolution bandwidth of 1 kHz. It is clear that the central frequency of $S(\Omega)$ in fig. S13(B) varies with v_r.

Notice that the frequency dependence of $S(\Omega)$ in fig. S13(B) is different from that in Fig. 4D. The visibility V_Ω extracted from fig. S13(B) varies with frequency Ω since the input is narrow band and the average photon number $\eta'\bar{n}'$ (see Eq. (11)) does not stay constant within the frequency response range of Q-RX. The whole spectrum of the narrowband input is located on the left side of ω_l (see the inset of Fig. 7A). Such an arrangement allows us to identify the moving direction of object from the accurate quantity of Doppler shift $f_d = \frac{2\omega_0 v_r}{c}$, but the cost is to tolerate the 3 dB signal attenuation because the symmetric field of the input located on right side of ω_l is vacuum.

To obtain the spectra in Fig.7B, we need to figure out the fringe size of interference pattern for each frequency component $\omega_l - \Omega$ with $\Omega = 2.9 + n \times \Delta f$ MHz ($0 \le n \le 200$ and $\Delta f = 1$ kHz) when v_r takes different value. In the experiment, $\Delta T_e - \Delta T = 0$, and the relative phase θ is slowly scanned at a rate of about 1.5 Hz. Figure S14 shows a set of raw data sampled from the current addition $i_+(t) = \langle \hat{\imath}_+(t) \rangle$ of two Q-RXs when the filter with passband of 2.9-3.1 MHz is applied in DSP. The orange (yellow) rectangle marks the data in the time window, which is equivalent to the current addition obtained by locking the phase to $\theta + \varphi_1 - \varphi_2 = 0$ $(\frac{\pi}{2})$ (see Eq. (10)). By applying FFT to the data points selected by the orange (yellow) rectangle, in principle, it is straight forward to obtain the optimum (minimum) average power $\langle {i_+}^2 \rangle_{max}$ ($\langle {i_+}^2 \rangle_{min}$) of interference pattern at the frequency $\Omega = 2.9 + n \times \Delta f$ MHz ($0 \le n \le 200$ and $\Delta f = 1$ kHz), with which the average photon number $\bar{n}'\eta'$ at different frequency can be calculated by eq. (s4). In our experiment, the back ground noise photon is negligible. So the valleys of interference fringe always overlap with shot noise level; the peaks of interference fringes at different frequency are obtained by FFT when the time window marked by orange is chosen to be 50 ms.

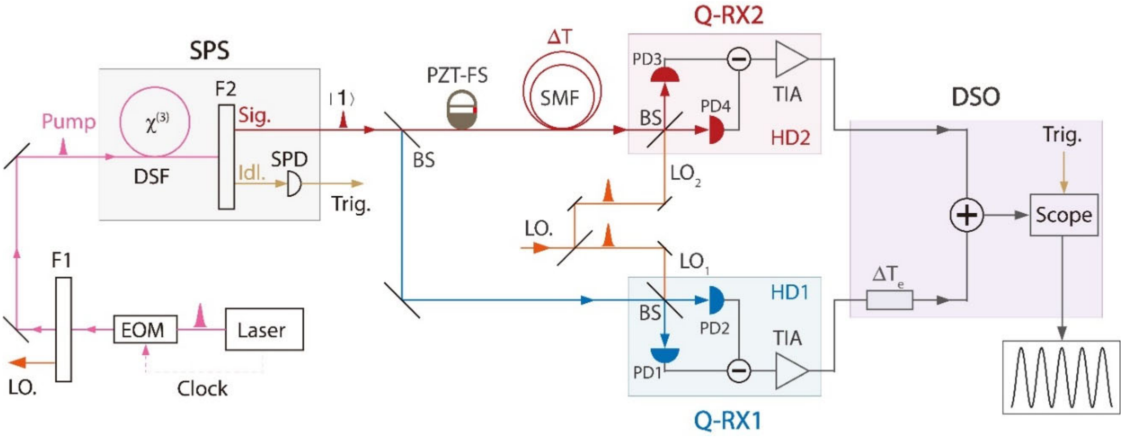

Fig. S1.

Experimental setup for observing quantum interference without optical superposition. Laser, mode locked fiber laser; EOM, electro-optic modulator; F1, dual band filter with pass bands centering at pump (1549.3 nm) and LO (1553.3 nm), respectively; SPS, single photon source; DSF, dispersion shifted fiber; F2, dual band filter with pass bands centering at heralding idler field (1545.3 nm) and heralded signal field (1553.3 nm), respectively; SPD, single photon detector; BS, 50/50 beam splitter; ΔT, optical delay; SMF, single mode fiber; PZT-FS, piezoelectric transducer driven fiber stretcher; LO, local oscillator; Q-RX, quantum receiver realized by homodyne detection (HD); PD, photodiode, TIA, transimpedance amplifier; ΔT_e, electronic delay; DSO, digital storage oscilloscope for digital signal processing.

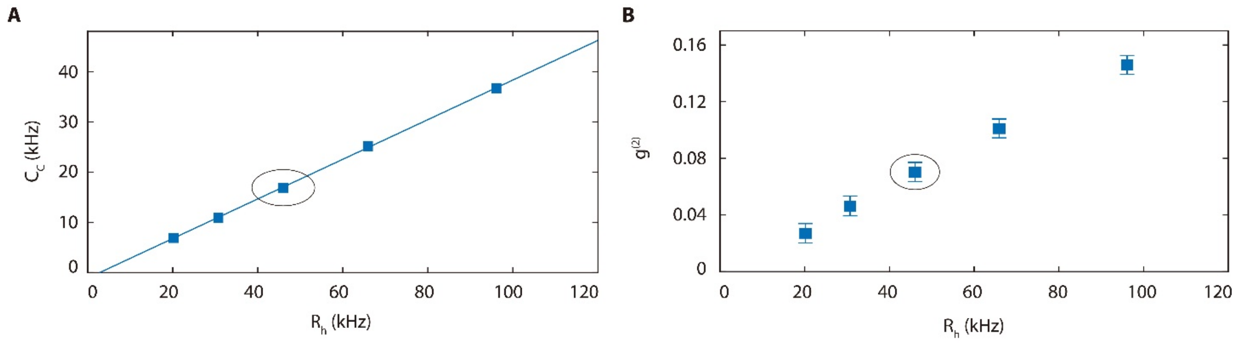

Fig. S2.

The heralding efficiency and photon statistics of the heralded single photon source. (**A**) Coincidence counting rate C_c between the heralded signal channel and heralding idler channel and (**B**) Intensity correlation $g^{(2)}$ of the heralded single photon source versus the counting rate of heralding idler channel, R_h. The solid line in plot (A) is the linear fit of the data points.

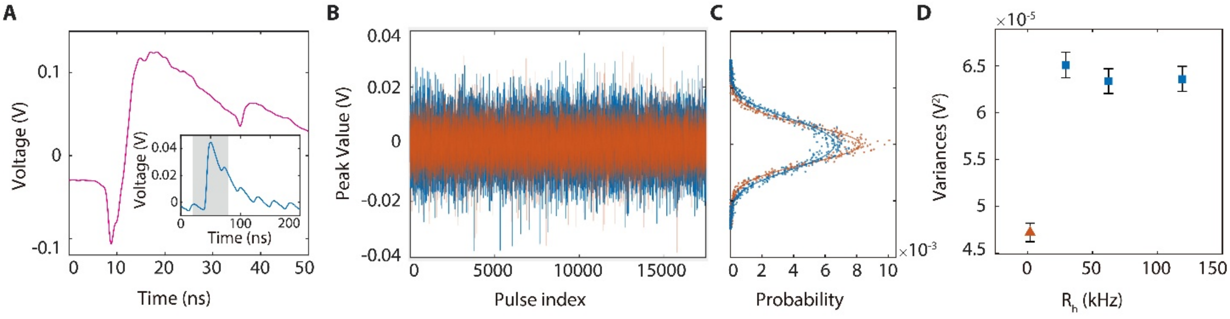

Fig. S3.

Results for measuring the amplitude probability distribution of heralded single photon source by homodyne detection (HD). (**A**) The typical trace of one electronic current pulse out of the balanced HD. The inset plots the normalized response function of one photodiode of homodyne detector. (**B**) Peak values of 17500 pulses out of HD for the input field of vacuum (orange trace) and the heralded single photons (blue trace), respectively. (**C**) The probability histogram of electrical pulses out of HD when the input is vacuum state (orange dots) and heralded single photons (blue dots), respectively. The solid curves are the theoretical fitting of the histogram. (**D**) Variances of the probability histogram when the input is vacuum (orange triangle) and heralded single photons (blue squares) with different heralding rate R_h, respectively. The variances of the histogram for single photon source with different heralding rate R_h are about 1.2 dB higher than that for vacuum state.

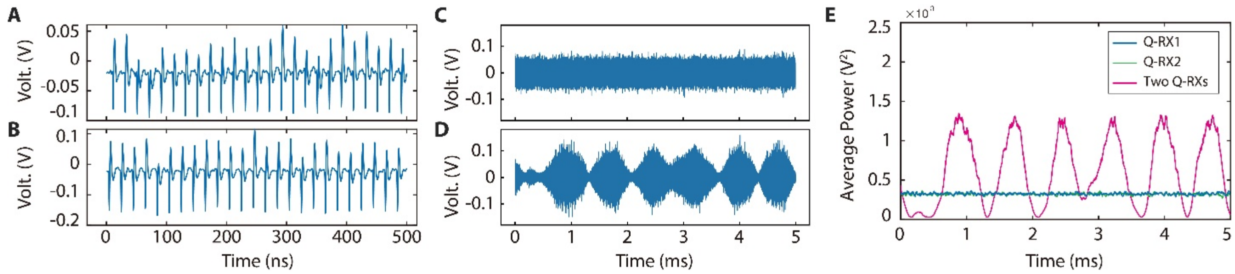

Fig. S4.

Data for recovering interference when the input of Fig. 1 is replaced with thermal state to mimic the photon statistics of star light and the two spatially distinct locations A and B is separated apart by a 1-km-long single mode fiber. (**A**) and (**B**): Raw data of the photo-currents (in terms of voltage) for individual quantum receiver (Q-RX) and current addition of two Q-RXs, respectively, in the time window of 500 ns. (**C**) and (**D**): The current of individual Q-RX $\langle \hat{\imath}_{HDi}(t) \rangle$ $(i = 1,2)$ and current addition of two Q-RXs $\langle \hat{\imath}_{+}(t) \rangle$, which are extracted from the peak of each electrical current pulse in (A) and (B), respectively. (**E**) The average powers for current addition of two Q-RXs $\langle \hat{\imath}_{+}^{2}(t) \rangle$ (pink trace) and current of individual Q-RXi $\langle \hat{\imath}_{HDi}{}^{2}(t) \rangle$ (blue and green traces for $(i = 1,2)$). The pink trace indicates the visibility of interference is about 94%, while no interference is observable from the green and blue traces. In the experiment, the relative phase between two optical paths is scanned by using piezoelectric transducer driven fiber stretcher (PZT-FS), the delay induced by optical path imbalance is $\Delta T \approx 5$ μs, and the total detection efficiency is about 30%. The average number, repetition rate and coherence time of thermal input field are $\bar{n}' \approx 27$ photons per pulse, 50 MHz and $\tau_c \approx 3$ ps, respectively.

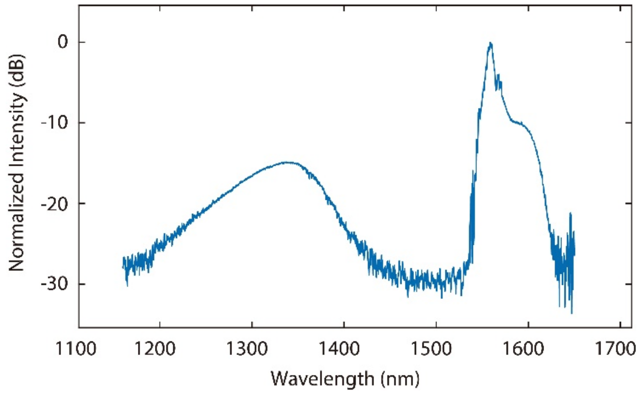

Fig. S5.
Spectrum of the broadband continuous wave thermal light source.

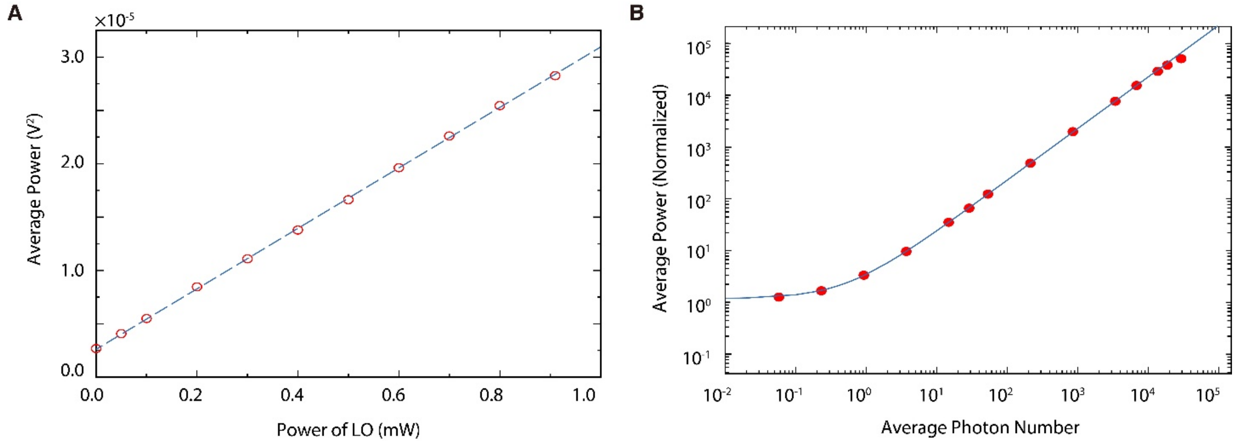

Fig. S6.

The linear dynamic range of individual quantum receiver (Q-RX) when the input is continuous wave broadband thermal light. (**A**) The shot noise level $\langle i^2 \rangle_{SNL}$ (in terms of voltage) measured by changing the power of local oscillator (LO) and blocking the input of Q-RX. The dashed line is obtained by fitting the data (red hollow circles) with eq. (S3). (**B**) The normalized average power of Q-RX versus the average photon number of input $\eta \bar{n}'$ when the power of LO is about 0.8 mW. The blue curve is obtained by fitting the data (red dots) with the eq. (S2): $\langle \hat{\imath}_{HD}{}^2(t) \rangle = \langle i^2 \rangle_{SNL}(2\eta\bar{n}' + 1)$, where $\langle i^2 \rangle_{SNL} = 2.64 \times 10^{-5}\, V^2$. In this measurement, the response time of Q-RX is $T_R \approx$ 10 ns, and the bandwidth of electrical filter applied in DSP is 100 MHz.

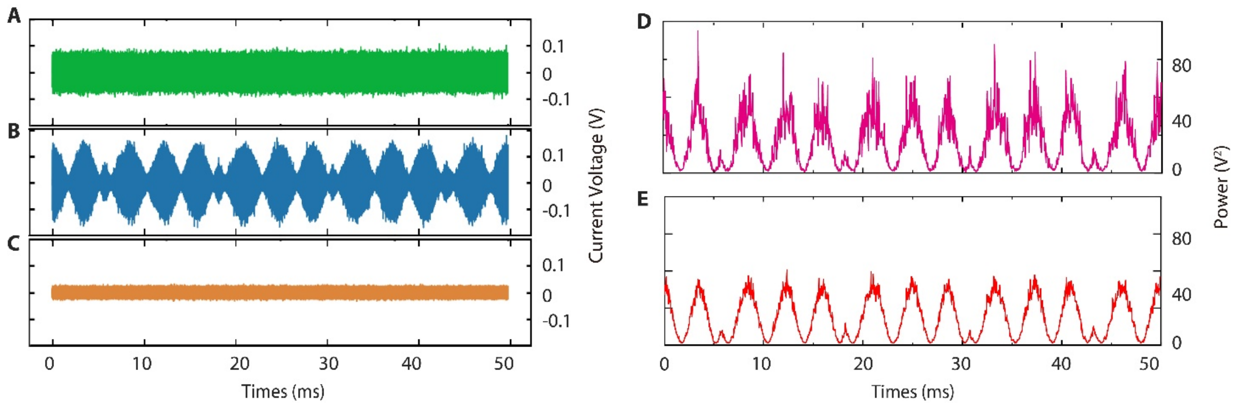

Fig. S7.

A typical set of data for recovering the interference and processing the interference fringes when the input of Fig. 4A is continuous wave broadband thermal light. Raw data sampled from (**A**) the photo-current $\langle \hat{\imath}_{HDi}(t) \rangle$ $(i = 1,2)$ (in terms of voltage) of individual quantum receiver (Q-RX) and (**B**) current addition $\langle \hat{\imath}_{+}(t) \rangle$ of two Q-RXs, respectively, when the average photon number of input is $\eta'\bar{n}' \approx 7$. (**C**) Raw data of shot noise level sampled from the current addition $\langle \hat{\imath}_{+}(t) \rangle_{SNL}$ by blocking the input of each Q-RX. (**D**) A typical fringe pattern in Fig. 4C for the input at the individual frequency $\omega_l + n \times 200$ kHz, where n is an integer in the range of $0 \le n \le 500$. (**E**) The interference fringe obtained by multiplexing 9 sets of fringe patterns in Fig. 4C, illustrating that the noise of interference fringe can be reduced by multiplexing the spectrally resolved interference patterns. In the experiment, $\lambda_l = \frac{2\pi c}{\omega_l} = 1550.1$ nm, $|\Delta T - \Delta T_e| = 0$, and the sampling rate in digital signal processing is 1.25 GHz.

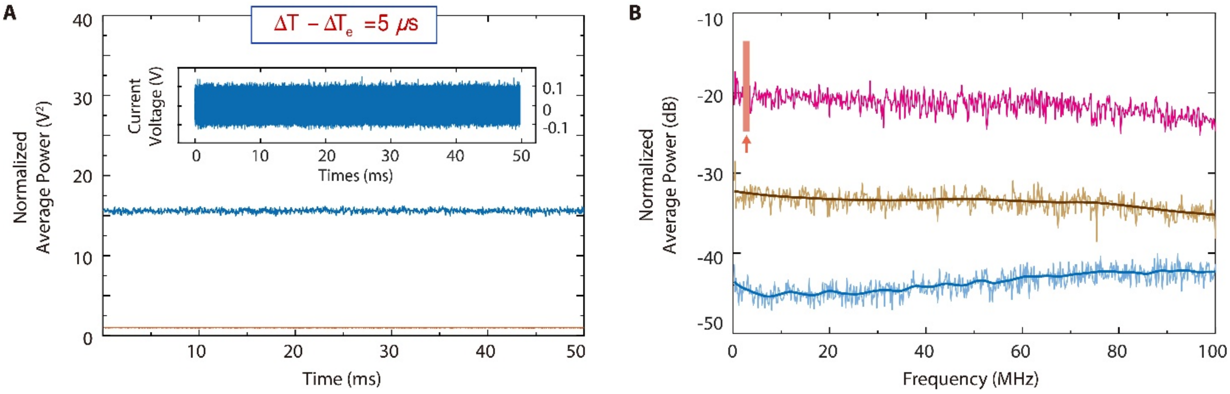

Fig. S8.

(A) The normalized average power and (B) power spectrum of current addition when $|\Delta T - \Delta T_e| = 5\mu s$ and the sampling rate in digital signal processing (DSP) is the same as in fig. S7. The interference is unobservable from the raw data sampled from the current addition$\langle \hat{\imath}_+(t) \rangle$ (inset in (A) and from the normalized average power of current addition $\langle \hat{\imath}_+^2(t) \rangle$ (in terms of voltage). The normalized power spectrum $S(\Omega)$ (pink trace) is obtained by using the algorithm of Fast Fourier Transform in DSP when the phase and frequency are scanned simultaneously. The dependence of visibility upon the frequency Ω is unobservable from the pink trace since the resolution bandwidth 200 kHz applied in DSP is too large to resolve the periodicity of $S(\Omega)$ (see Eq. (11)). The shot noise level (brown trac) of $S(\Omega)$ is obtained by blocking the input field of two Q-RXs. The electronic noise level (light blue trace) in analyzing $S(\Omega)$ is obtained by blocking the input and LO of each Q-RX. The range marked by the orange arrow and thick orange line is 1.8-2 MHz. In the experiment, the average photon number of input is $\bar{n}'\eta' \approx 7$, and the response time of each Q-RX is $T_R \approx$ 10 ns. The orange line in (A) represents the shot noise level. The three traces in (B) are normalized to $1V^2$.

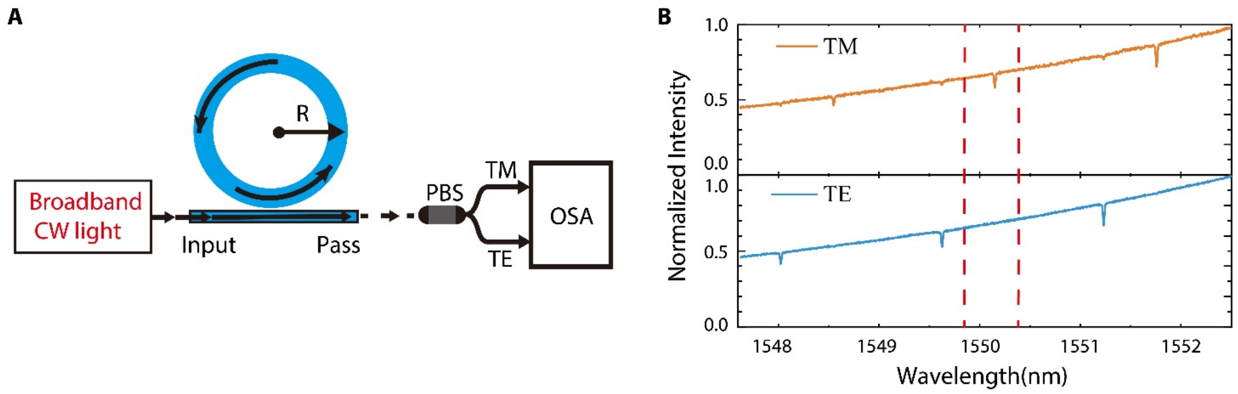

Fig. S9.

All-pass ring resonator and its transmission spectrum. (**A**) Experimental setup for testing the spectrum of micro-ring resonator side-coupled to a bus waveguide. The broadband continuous wave (CW) thermal light (in fig. S5) is launched to the input port. The polarization beam splitter (PBS) in front of the optical spectrum analyzer (OSA) is used to selected the transmission light in TE and TM modes. (**B**) Part of the whole spectrum in TE and TM modes. The dips around the ring resonances wavelength are observable in both TE and TM modes. The details of the range marked by two dashed red lines is demonstrated in fig. S10 and Fig. 5. In the experiment, the radius of micro-ring resonator is $R = 135$ μm, and the resolution of OSA is 0.02 nm.

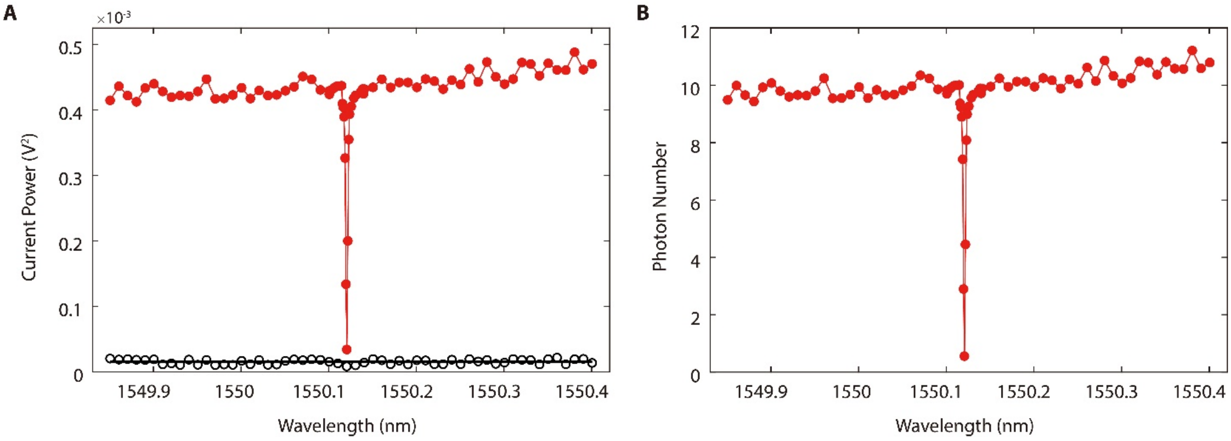

Fig. S10.

The spectrum of absorption line measured by the new interferometer when the wavelength of local oscillator (LO) is varied. (**A**) The size of interference fringes versus the wavelength of LO. The red solid (black hollow) circles represent the maximum (minimum) of the interference fringes recovered from the average power of current addition of two quantum receivers $\langle \hat{\imath}_{+}^{2}(t)\rangle$. The shot noise level (black line) is $\langle i^2\rangle_{SNL} = 8.9 \times 10^{-6}\, V^2$ (**B**) Average photon number $\eta'\bar{n}'$ as a function of wavelength for the input field. In the experiment, the polarization of LO is properly adjusted to select the TM mode of absorption medium, the bandwidth of electrical filter applied in DSP is 10 MHz, and the tunning step of the wavelength of LO is 0.01 nm.

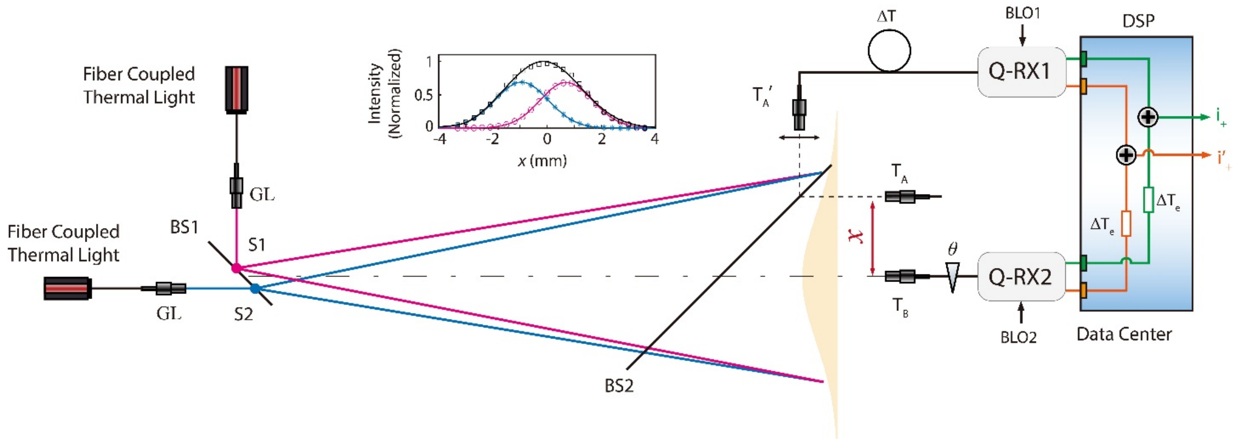

Fig. S11.

Experimental setup of the new interferometer for measuring the distance between two independent incoherent point sources (S1, S2). GL, grin lens; BS, 50/50 beam splitter, T_A ,T_B, T_A', fiber coupled lenses with diameter of 0.9 mm; ΔT, the optical path imbalance induced delay due to the unequal fiber patch cords connected with T_A' and T_B; Q-RX1, Q-RX2, quantum receivers; BLO, bichromatic local oscillator realized by combining two single frequency lasers at λ_l and λ_l', respectively; DSP, digital signal processing. T_A' and T_A are mirror images of each other. T_B with equal distance between S1 and S2 is placed along the optical axis represented by dash-dotted line, and the distance x between T_A and T_B is the baseline length of the interferometer. The yellow shade illustrates the intensity distribution of light (described by the black trace and squares in inset) one meter away from S1 and S2. The inset is the intensity of light collected by individual T_A' at different position when only one point source (red circles for S1 and blue stars for S2) and both sources (black squares) are turned on, respectively. The solid curves in the inset are the fitting to a Gaussian function.

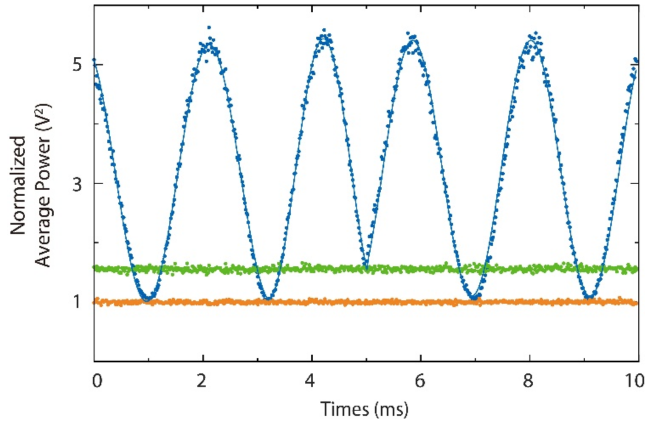

Fig. S12.

A typical set of interference pattern for the input the fields selected by local oscillators with wavelength $\lambda_l{}'$ =1605 nm. The interference is recovered from the normalized power (in terms of voltage) of current addition $\langle \hat{\imath}'^2_+(t) \rangle$ (blue dots) of two quantum receivers (Q-RXs). The normalized average powers of individual Q-RX (green dots) stay constant, illustrating the point sources S1 and S2 are independent. The normalizations of the average powers are done to the shot noise level (orange trace) $\langle \hat{\imath}^2_+ \rangle_{SNL} = 5.68 \times 10^{-5}$ V^2. The fitting (blue curve) indicates the visibility is about 67%. In the experiment, the baseline length in fig. 6A is $x = 0$, the average photon number of input is $\eta'\bar{n}' \approx 1$, and $\Delta T_e - \Delta T = 0$.

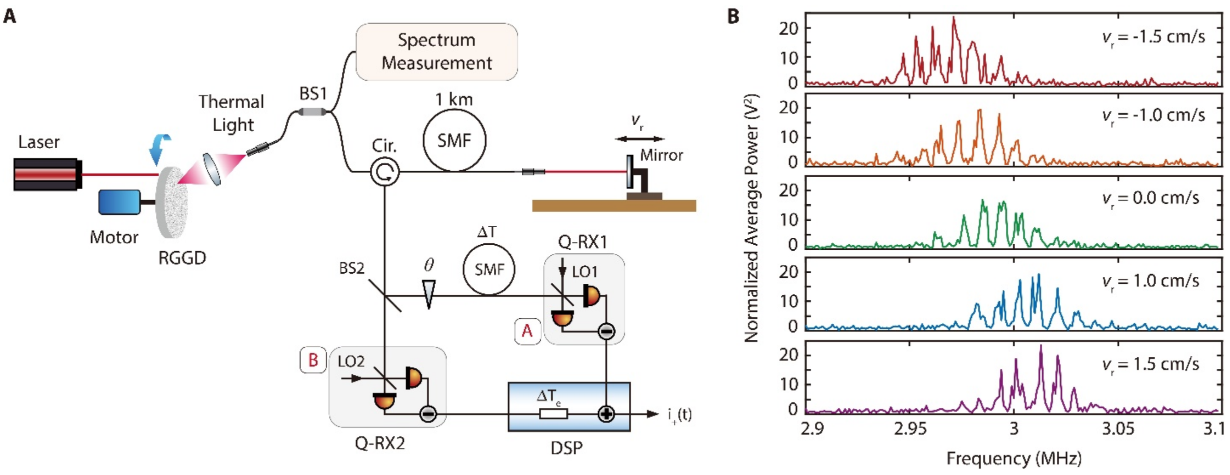

Fig. S13.

(A) The experimental setup and (B) results of power spectrum $S(\Omega)$ normalized to shot noise level for estimating the Doppler shift induced by the radial velocity v_r of a moving object. RGGD, rotating ground glass disk; BS, 50/50 beam splitter; Cir, circulator used to direct the light reflected off the mirror into the new interferometer; ΔT, optical delay; SMF, single mode fiber; LO, local oscillator; Q-RX, quantum receiver; ΔT_e, electronic delay; DSP, digital signal processing. In the experiment, ΔT_e −ΔT=0, the intensity of light collected at the location A (or B) is about 4×10^5 photons/s.

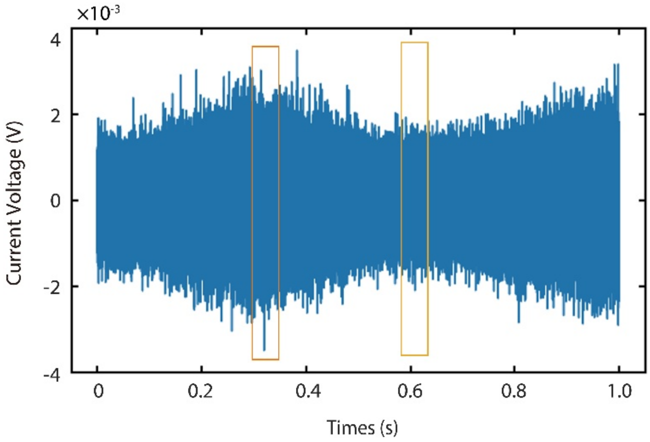

Fig. S14.

A typical set of raw data sampled from the current addition of two Q-RXs when the relative phase θ in fig. S13 is slowly scanned. The rectangles in orange and yellow respectively mark the time windows in the vicinity of relative phase $\theta + \varphi_1 - \varphi_2 = 0, \frac{\pi}{2}$ (see Eq. (10)), at which the constructive and destructive interference occurs. In the experiment, the intensity of reflected light at the location A (or B) is about 4×10^5 photons/s, $\Delta T_e - \Delta T=0$, the bandpass electric filter of 2.9-3.1 MHz is applied in DSP, and the sampling rate is 100 MHz.

REFERENCES

1. K. G. Jansky, Radio waves from outside the solar system. *Nature* **132**, 66–66 (1933).

2. M. Ryle, A. Hewish, The synthesis of large radio telescopes. *Mon. Not. R. Astron. Soc.* **120**, 220–230 (1960).

3. A. R. Thompson, J. M. Moran, G. W. Swenson, *Interferometry and Synthesis in Radio Astronomy* (Springer, ed. 3, 2017).

4. Y. Asaki, B. A. Pampliega, P. G. Edwards, S. Iguchi, E. J. Murphy, Astronomical radio interferometry. *Nat. Rev. Methods Primers* **3**, 89 (2023).

5. J. D. Monnier, Optical interferometry in astronomy. *Rep. Prog. Phys.* **66**, 789–857 (2003).

6. M. A. Johnson, A. L. Betz, C. H. Townes, 10-μm heterodyne stellar interferometer. *Phys. Rev. Lett.* **33**, 1617–1620 (1974).

7. D. D. S. Hale, M. Bester, W. C. Danchi, W. Fitelson, S. Hoss, E. A. Lipman, J. D. Monnier, P. G. Tuthill, C. H. Townes, The Berkeley infrared spatial interferometer: A heterodyne stellar interferometer for the mid-infrared. *Astrophys. J.* **537**, 998–1012 (2000).

8. D. Gottesman, T. Jennewein, S. Croke, Longer-baseline telescopes using quantum repeaters. *Phys. Rev. Lett.* **109**, 070503 (2012).

9. M. R. Brown, M. Allgaier, V. Thiel, J. D. Monnier, M. G. Raymer, B. J. Smith, Interferometric imaging using shared quantum entanglement. *Phys. Rev. Lett.* **131**, 210801 (2023).

10. X. Tang, Y. Zhang, X. Guo, L. Cui, X. Li, Z. Y. Ou, Phase-dependent Hanbury-Brown and Twiss effect for the complete measurement of the complex coherence function. *Light Sci. Appl.* **14**, 46 (2025).

11. I. Newton, *Opticks: Or A Treatise of the Reflexions, Refractions, Inflexions and Colours of Light* (Royal Society,1704).

12. G. Kirchhoff, R. Bunsen, Chemical analysis by observation of spectra. *Ann. Phys. Chem.* **110**, 598–625 (1860).

13. I. Appenzeller, *Introduction to Astronomical Spectroscopy* (Cambridge Univ. Press, 2013).

14. M. Mayor, D. Queloz, A Jupiter-mass companion to a solar-type star. *Nature* **378**, 355–359 (1995).

15. M. Mayor, C. Lovis, N. C. Santos, Doppler spectroscopy as a path to the detection of earth-like planets. *Nature* **513**, 328–335 (2014).

16. J. Liske, A. Grazian, E. Vanzella, M. Dessauges, M. Viel, L. Pasquini, M. Haehnelt, S. Cristiani, F. Pepe, G. Avila, P. Bonifacio, F. Bouchy, H. Dekker, B. Delabre, S. D'Odorico, V. D'Odorico, S. Levshakov, C. Lovis, M. Mayor, P. Molaro, L. Moscardini, M. T. Murphy, D. Queloz, P. Shaver, S. Udry, T. Wiklind, S. Zucker, Cosmic dynamics in the era of extremely large telescopes. *Mon. Not. R. Astron. Soc.* **386**, 1192–1218 (2008).

17. A. F. Goetz, G. Vane, J. E. Solomon, B. N. Rock, Imaging spectrometry for earth remote sensing. *Science* **228**, 1147–1153 (1985).

18. A. Zahra, R. Qureshi, M. Sajjad, F. Sadak, M. Nawaz, H. Ahmad Khan, M. Uzair, Current advances in imaging spectroscopy and its state-of-the-art applications. *Expert Syst. Appl.* **238**, 122172 (2024).

19. A. Wilkinson, R. M. Sharpless, A. E. M. Fosbury, P. T. Wallace, Stellar dynamics of Cen A. *Mon. Not. R. Astron. Soc.* **218**, 297–329 (1986).

20. J. Mariotti, S. T. Ridgway, Double Fourier spatio-spectral interferometry—Combining high spectral and high spatial resolution in the near infrared. *Astron. Astrophys.* **195**, 350–363 (1988).

21. E. Mediavilla, S. Arribas, M. M. Roth, J. Cepa-Nogué, F. Anchez, Eds. *3D Spectroscopy in Astronomy* (Cambridge Univ. Press, 2022).

22. A. A. Michelson, F. G. Pease, Measurement of the diameter of alpha-orionis by the interferometer. *Astrophys. J.* **7**, 143–146 (1921).

23. P. Lawson, *Principles of Long Baseline Stellar Interferometry: Course Notes from the 1999 Michelson Summer School* (JPL publication, 2000).

24. H. P. Yuen, V. W. S. Chan, Noise in homodyne and heterodyne detection. *Opt. Lett.* **8**, 177–179 (1983).

25. J. Shapiro, H. Yuen, J. Machado Mata, Optical communication with two-photon coherent states–Part II: Photoemissive detection and structured receiver performance. *IEEE Trans. Inf. Theory* **25**, 179–192 (1979).

26. Z. Y. Ou, S. F. Pereira, H. J. Kimble, K. C. Peng, Realization of the Einstein-Podolsky-Rosen paradox for continuous variables. *Phys. Rev. Lett.* **68**, 3663 (1992), 3666.

27. H. J. Kimble, M. Dagenais, L. Mandel, Photon antibunching in resonance fluorescence. *Phys. Rev. Lett.* **39**, 691–695 (1977).

28. B. Burke, Quantum interference paradox. *Nature* **223**, 389–390 (1969).

29. M. A. Johnson, C. H. Townes, Quantum effects and optimization of heterodyne detection. *Opt. Commun.* **179**, 183–187 (2000).

30. P. A. M. Dirac, *The Principles of Quantum Mechanics* (Clarendon Press, ed. 4, 1981).

31. G. I. Taylor, Interference fringes with feeble light. *Proc. Camb. Philos. Soc.* **15**, 114–115 (1909).

32. P. Grangier, G. Roger, A. Aspect, Experimental evidence for a photon anticorrelation effect on a beam splitter: A new light on single-photon interferences. *Europhys. Lett.* **1**, 173–179 (1986).

33. N. Bohr, *Quantum Theory and Measurement* (Princeton Univ. Press, 1983).

34. J. K. Webb, J. A. King, M. T. Murphy, V. V. Flambaum, R. F. Carswell, M. B. Bainbridge, Indications of a spatial variation of the fine structure constant. *Phys. Rev. Lett.* **107**, 191101 (2011).

35. M. Taylor, Coherent detection method using DSP for demodulation of signal and subsequent equalization of propagation impairment. *IEEE Photonics Technol. Lett.* **16**, 674–676 (2004).

36. N. Huo, L. Cui, Y. Zhang, W. Zhao, X. Guo, Z. Y. Ou, X. Li, Measurement-dependent erasure of distinguishability for the observation of interference in an unbalanced SU(1,1) interferometer. *PRX Quantum* **3**, 020313 (2022).

37. Y. Zhang, X. Tang, X. Guo, L. Cui, X. Li, Z. Y. Ou, Optical interference by amplitude measurement. *Phys. Rev. Res.* **7**, 013255 (2025).

38. K. Kikuchi, Fundamentals of coherent optical fiber communications. *J. Lightwave Technol.* **34**, 157–179 (2016).

39. S. M. Tan, D. F. Walls, M. J. Collett, Nonlocality of a single photon. *Phys. Rev. Lett.* **66**, 252–255 (1991).

40. L. Yang, X. Ma, X. Guo, L. Cui, X. Li, Characterization of a fiber-based source of heralded single photons. *Phys. Rev. A* **83**, 053843 (2011).

41. J. Li, J. Su, L. Cui, T. Xie, Z. Y. Ou, X. Li, Generation of pure-state single photons with high heralding efficiency by using a three-stage nonlinear interferometer. *Appl. Phys. Lett.* **116**, 204002 (2020).

42. S. A. Castelletto, R. E. Scholten, Heralded single photon sources: A route towards quantum communication technology and photon standards. *Eur. Phys. J. Appl. Phys.* **41**, 181–194 (2008).

43. A. I. Lvovsky, Iterative maximum-likelihood reconstruction in quantum homodyne tomography. *J. Opt. B: Quantum Semiclass. Opt.* **6**, S556–S559 (2004).

44. N. Liu, Y. Liu, X. Guo, L. Yang, X. Li, Z. Y. Ou, Approaching single temporal mode operation in twin beams generated by pulse pumped high gain spontaneous four wave mixing. *Opt. Express* **24**, 1096–1108 (2016).

45. E. Obrzud, M. Rainer, A. Harutyunyan, M.H. Anderson, M. Geiselmann, B. Chazelas, S. Kundermann, S. Lecomte, M. Cecconi, A. Ghedina, E. Molinari, F. Pepe, F. Wildi, F. Bouchy, T.J. Kippenberg, T. Herr, A microphotonic astrocomb. *Nat. Photonics* **13**, 31–35 (2019).

46. C. Bare, B. G. Clark, K. I. Kellermann, M. H. Cohen, D. L. Jauncey, Interferometer experiment with independent local oscillators. *Science* **157**, 189–191 (1967).

47. E. Oelker, R. B. Hutson, C. J. Kennedy, L. Sonderhouse, T. Bothwell, A. Goban, D. Kedar, C. Sanner, J. M. Robinson, G. E. Marti, D. G. Matei, T. Legero, M. Giunta, R. Holzwarth, F. Riehle, U. Sterr and J. Ye, Demonstration of 4.8×10^{-17} stability at 1 s for two independent optical clocks. *Nat. Photonics* **13**, 714–719 (2019).

48. K. Predehl, G. Grosche, S. M. F. Raupach, S. Droste, O. Terra, J. Alnis, T. Legero, T. W. Hänsch, T. Udem, R. Holzwarth, H. Schnatz, A 920-kilometer optical fiber link for frequency metrology at the 19th decimal place. *Science* **336**, 441–444 (2012).

49. Q. Shen, J. Guan, J. Ren, T. Zeng, L. Hou, M. Li, Y. Cao, J. Han, M. Lian, Y. Chen, X. Peng, S. Wang, D. Zhu, X. Shi, Z. Wang, Y. Li, W. Liu, G. Pan, Y. Wang, Z. Li, J. Wu, Y. Zhang, F. Chen, C. Lu, S. Liao, J. Yin, J. Jia, C. Peng, H. Jiang, Q. Zhang, J. Pan, Free-space dissemination of time and frequency with 10^{-19} instability over 113 km. *Nature* **610**, 661–666 (2022).

50. T. Udem, R. Holzwarth, T. W. Hänsch, Optical frequency metrology. *Nature* **416**, 233–237 (2002).

51. R. A. Probst, D. Milaković, B. Toledo-Padrón, G. L. Curto, G. Avila, A. Brucalassi, B. L. C. Martins, I. Leão, M. Esposito, J. Hernández, F. Grupp, T. W. Hänsch, H. Kellermann, F. Kerber, O. Mandel, A. Manescau, E. Pozna, R. Rebolo, J. Medeiros, T. Steinmetz, A. Mascareño, T. Udem, J. Urrutia, Y. Wu, L. Pasquini, R. Holzwarth, A crucial test for astronomical spectrograph calibration with frequency combs. *Nat. Astron.* **4**, 603–608 (2020).

52. R. Hanbury-Brown, R. Q. Twiss, Correlation between photons in two coherent beams of light. *Nature* **177**, 27–29 (1956).

53. W. Bogaerts, P. DeHeyn, T. V. Vaerenbergh, K. DeVos, S. K. Selvaraja, T. Claes, P. Dumon, P. Bienstman, D. V. Thourhout, R. Baets, Silicon microring resonators. *Laser Photonics Rev.* **6**, 47–73 (2012).

54. M. Ferrera, D. Duchesne, L. Razzari, M. Peccianti, R. Morandotti, P. Cheben, S. Janz, D.-X. Xu, B. E. Little, S. Chu, D. J. Moss, Low power four wave mixing in an integrated, micro-ring resonator with Q = 1.2 million. *Opt. Express* **17**, 14098–14103 (2009).
